# Mitochondrial optic neuropathies – Disease mechanisms and therapeutic strategies

**DOI:** 10.1016/j.preteyeres.2010.11.002

**Published:** 2011-03

**Authors:** Patrick Yu-Wai-Man, Philip G. Griffiths, Patrick F. Chinnery

**Affiliations:** a Mitochondrial Research Group, Institute for Ageing and Health, The Medical School, Newcastle University, UK; b Department of Ophthalmology, Royal Victoria Infirmary, Newcastle upon Tyne, UK; c Institute of Human Genetics, Newcastle University, Newcastle upon Tyne NE1 3BZ, UK

**Keywords:** Dominant optic atrophy, Glaucoma, Hereditary spastic paraplegia, Leber hereditary optic neuropathy, Mitochondrial DNA, Mitofusin, Multiple sclerosis, Neuroprotection, Optic neuritis, Optic neuropathy, Retinal ganglion cell

## Abstract

Leber hereditary optic neuropathy (LHON) and autosomal-dominant optic atrophy (DOA) are the two most common inherited optic neuropathies in the general population. Both disorders share striking pathological similarities, marked by the selective loss of retinal ganglion cells (RGCs) and the early involvement of the papillomacular bundle. Three mitochondrial DNA (mtDNA) point mutations; m.3460G>A, m.11778G>A, and m.14484T>C account for over 90% of LHON cases, and in DOA, the majority of affected families harbour mutations in the *OPA1* gene, which codes for a mitochondrial inner membrane protein. Optic nerve degeneration in LHON and DOA is therefore due to disturbed mitochondrial function and a predominantly complex I respiratory chain defect has been identified using both *in vitro* and *in vivo* biochemical assays. However, the trigger for RGC loss is much more complex than a simple bioenergetic crisis and other important disease mechanisms have emerged relating to mitochondrial network dynamics, mtDNA maintenance, axonal transport, and the involvement of the cytoskeleton in maintaining a differential mitochondrial gradient at sites such as the lamina cribosa. The downstream consequences of these mitochondrial disturbances are likely to be influenced by the local cellular *milieu*. The vulnerability of RGCs in LHON and DOA could derive not only from tissue-specific, genetically-determined biological factors, but also from an increased susceptibility to exogenous influences such as light exposure, smoking, and pharmacological agents with putative mitochondrial toxic effects. Our concept of inherited mitochondrial optic neuropathies has evolved over the past decade, with the observation that patients with LHON and DOA can manifest a much broader phenotypic spectrum than pure optic nerve involvement. Interestingly, these phenotypes are sometimes clinically indistinguishable from other neurodegenerative disorders such as Charcot-Marie-Tooth disease, hereditary spastic paraplegia, and multiple sclerosis, where mitochondrial dysfunction is also thought to be an important pathophysiological player. A number of vertebrate and invertebrate disease models has recently been established to circumvent the lack of human tissues, and these have already provided considerable insight by allowing direct RGC experimentation. The ultimate goal is to translate these research advances into clinical practice and new treatment strategies are currently being investigated to improve the visual prognosis for patients with mitochondrial optic neuropathies.

## 1 Introduction

Inherited optic neuropathies affect at least 1 in 10,000 individuals and as a group, they represent an important cause of chronic visual impairment (Man et al., 2003; Newman and Biousse, 2004; Schaefer et al., 2008; Yu-Wai-Man et al., 2010a). Historically, these inherited optic nerve disorders were classified according to their mode of inheritance, and whether they were isolated or part of a more complicated syndromal variant. The identification of the underlying genetic defects in a large number of these inherited optic neuropathies now allows for a more accurate molecular classification, which has greatly benefited diagnostic accuracy and genetic counselling. The two classical prototypes are Leber hereditary optic neuropathy (LHON) and autosomal-dominant optic atrophy (DOA), which are both characterised by the preferential loss of retinal ganglion cells (RGCs) (Carelli et al., 2009). LHON is due to primary mitochondrial DNA (mtDNA) mutations, whereas the majority of patients with DOA harbour pathogenic mutations within the *OPA1* gene, which codes for a mitochondrial inner membrane protein (Yu-Wai-Man et al., 2009b; Fraser et al., 2010). As the genetic basis for other inherited optic neuropathies were uncovered, it became apparent that mitochondrial dysfunction is a recurrent molecular theme underlying the loss of RGCs in these disorders. In this review, we will cover basic aspects of mitochondrial biology and genetics, and how disruption of these critical biological systems contributes to optic nerve degeneration in different mitochondrial disease models.

## 2 The mitochondria

### 2.1 Evolutionary origin

Mitochondria are ubiquitous intracellular organelles and they fulfil a fundamental role by providing most of the adenosine triphosphate (ATP) requirements of eukaryotic cells (DiMauro and Schon, 2003). The prevailing endosymbiotic hypothesis suggests that mitochondria evolved from aerobic α-proteobacteria, which were then gradually assimilated by primitive glycolytic eubacteria in a symbiotic relationship (Margulis, 1971; Gray et al., 1999). During evolution, the α-proteobacteria gradually transferred the majority of their genetic material to the eubacteria’s nuclear chromosomes, creating the prototypal eukaryotic cell (Gabaldon and Huynen, 2004). Phylogenetic comparison of mtDNA between modern humans and other organisms, including *Rickettsia prowazekii*, an α-proteobacterium, supports this common evolutionary origin for the mitochondrial genome (Martin and Muller, 1998; Gray et al., 1999).

### 2.2 Structure

Mitochondria are tubular-shaped organelles bounded by an outer and an inner membrane, and these delineate two distinct compartments: an intermembrane space and an internal matrix space (Frey and Mannella, 2000). The outer membrane allows passive diffusion of low molecular weight molecules up to 10 kDa, and this permeability is conferred by a family of channel-forming proteins known as porins, or voltage dependent anion channels (VDAC). The inner membrane is highly convoluted and these multiple infoldings, known as cristae, greatly increase its effective surface area (Perkins et al., 1997). Compared to the outer membrane, the inner membrane is relatively impermeable except for specific active transport channels, allowing an electrochemical gradient to be established across this barrier. The inner membrane also contains a number of highly specialised proteins, including the respiratory chain complexes and members of the mitochondrial membrane protease family. The mitochondrial matrix compartment contains mtDNA molecules packaged within nucleoid structures, and it is also the site of multiple metabolic pathways essential for normal cellular function: the citric acid cycle, β-oxidation of fatty acids, steroid, amino acid, and pyrimidine biosynthesis (Raha and Robinson, 2000).

### 2.3 Oxidative phosphorylation

**Fig. 1:**
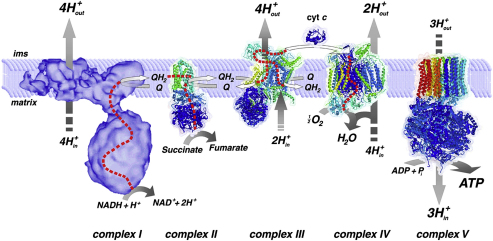
The mitochondrial respiratory chain and oxidative phosphorylation. Reproduced with permission from Nijtmans et al. (2004).

The mitochondrial respiratory chain comprises four multi-subunit polypeptide complexes (I–IV) which are embedded within the inner mitochondrial membrane (Fig. 1). The production of ATP is tightly regulated and it is the end-product of a process known as oxidative phosphorylation (OXPHOS). Acetyl-CoA, an intermediate product of glycolysis and β-oxidation, is metabolised further by the citric acid cycle to the reducing equivalents nicotinamide adenine dinucleotide hydrogen (NADH) and flavin adenine dinucleotide hydrogen (FADH_2_) (DiMauro and Schon, 2003). NADH and FADH_2_ are then re-oxidised by donating electrons to complexes I and II respectively. The energy released by the shuttling of these high energy electrons along the respiratory chain complexes allows protons to be pumped from the matrix compartment into the intermembrane space. Two additional carriers, ubiquinone (Co-enzyme Q_10_) and cytochrome *c*, also play critical roles in the efficient transfer of electrons through these successive oxidation–reduction reactions. The electrochemical gradient generated across the inner mitochondrial membrane is then used by complex V (ATP synthase) to catalyze the conversion of adenosine diphosphate (ADP) and inorganic phosphate (Pi) to ATP (Yoshida et al., 2001).

### 2.4 Mitochondrial genetics

**Fig. 2:**
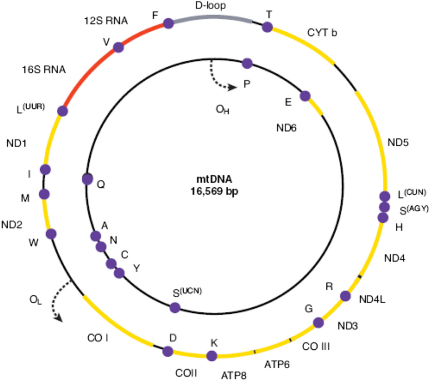
The human mitochondrial genome. Protein coding (yellow), rRNA (red), and tRNA (purple) genes are depicted on the heavy (H-, outer) and light (L-, inner) strands. The 22 tRNAs are indicated by their cognate amino acid letter code and the 2 rRNAs by their sedimentation coefficients (12S and 16S). The origins of mtDNA replication and the direction of synthesis are denoted by O_H_ for the H-strand, and O_L_ for the L-strand.

Mitochondria are unique in having their own genome in the form of a circular, double-stranded molecule 16,569 bp long (Fig. 2) (Anderson et al., 1981; Andrews et al., 1999b). It is a high-copy number genome with hundreds to thousands of mtDNA molecules per cell, depending on their specific energy requirements. MtDNA replicates continuously and this process is independent of nuclear genome replication, occurring in both mitotic and post-mitotic cells. The mitochondrial genome codes for 2 ribosomal RNAs (12S and 16S rRNA), 22 transfer RNAs (tRNAs), and 13 polypeptide subunits of the respiratory chain complexes. The majority of these genes is located on the H-strand, with only *MTND6* and eight tRNA genes found on the L-strand (Taylor and Turnbull, 2005). MtDNA is a very compact molecule with overlapping gene regions devoid of introns, and a small 1.1 kb non-coding region, known as the D-loop, which is involved in mtDNA transcription and replication.

### 2.5 Mitochondrial haplogroups

The mitochondrial genome accumulates mutations at a significantly faster rate compared to the nuclear genome, and several factors contribute to this higher mutational rate: the absence of protective histones, the lack of effective repair mechanisms, the high mtDNA replication rate increasing the likelihood of errors, and the close proximity of mtDNA molecules to the respiratory chain complexes where they are exposed to high levels of reactive oxygen species (ROS) (Howell et al., 1996; Jazin et al., 1998; Raha and Robinson, 2000). The mutational rate varies between different mtDNA regions, and it is much faster within the two hypervariable regions (HVR I and II) of the D-loop where a mutation is estimated to occur every 30 maternal generations (Parsons et al., 1997; Siguroardottir et al., 2000). MtDNA is therefore highly polymorphic, and during human evolution, a number of relatively benign mitochondrial sequence variants have become fixed in different populations. As mtDNA is maternally inherited, these polymorphisms have accumulated sequentially along radiating female lineages, following the pattern of human migration from Africa into the various continents some 150,000 years ago (Cann, 2001). The human phylogenetic tree contains 18 major mtDNA haplogroups, and these comprise a total of 497 haplogroup-defining polymorphic variants (Torroni and Wallace, 1994; Herrnstadt et al., 2002). Individuals of European ancestry belong to one of nine haplogroups: H, I, J, K, T, U, V, W and X, with haplogroup H accounting for nearly half of all cases.

### 2.6 Heteroplasmy and mutational threshold

There are about 10,000 mtDNA molecules per cell, with each mitochondrion containing multiple copies. Two possible situations can therefore arise, known as homoplasmy and heteroplasmy (Lightowlers et al., 1997; Chinnery, 2002). In the heteroplasmic state, two or more mtDNA variants are present at a specific nucleotide position, and the same phenomenon can also occur for mtDNA re-arrangements such as deletions. Most mtDNA mutations are heteroplasmic, a feature which supports the concept of a mutational threshold for pathogenicity (Chinnery et al., 2000; Taylor and Turnbull, 2005). The relationship between mutational load and respiratory chain activity has been extensively investigated in different tissues, and the deleterious consequences of most mtDNA mutations on OXPHOS usually become apparent when the proportion of the mutant species exceeds 60–80% (Shoubridge et al., 1990; Bua et al., 2006; Durham et al., 2007). There are mutation- and tissue-specific variations in this biochemical threshold (Corral-debrinski et al., 1992; Chinnery et al., 1999; Wang et al., 2001; Nekhaeva et al., 2002), and although these could account for the pattern of organ involvement and clinical severity associated with a particular mtDNA defect, the molecular mechanisms are likely to be much more complex.

### 2.7 Nuclear-mitochondrial interactions

**Fig. 3:**
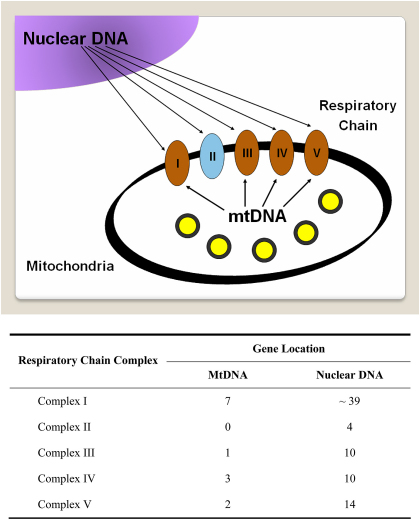
Mitochondrial and nuclear-encoded subunits of the mitochondrial respiratory chain complexes.

Mitochondria only have limited autonomy and they rely heavily on the nuclear genome for the majority of their structural and functional subunits (Fig. 3). Mitochondrial disorders can therefore arise secondary to both primary mtDNA mutations and nuclear genetic defects which disrupt mitochondrial-related proteins. In 2001, the first nuclear genes, *POLG1* and *PEO1*, were identified among families with autosomal-dominant chronic progressive external ophthalmoplegia (CPEO) associated with multiple mtDNA deletions (Spelbrink et al., 2001; Van Goethem et al., 2001). Since then, the number of genes causing nuclear mitochondrial disorders has expanded continuously (Table 1), allowing significant progress to be made in elucidating the fundamental mechanisms that underpin mitochondrial physiology in both normal and disease states. As a result, we have gained a better understanding of the complex interactions between subunits of the respiratory chain complexes, and the crucial role played by accessory proteins in ensuring their proper assembly and stability along the inner mitochondrial membrane. These sometimes rare neurodegenerative and metabolic disorders have also provided important insights into the molecular components required for mtDNA maintenance, and the translational machinery that regulates intra-mitochondrial protein synthesis.

**Table 1:** Nuclear mitochondrial disorders.

*Mutations involving structural subunits of the mitochondrial respiratory chain*
Leigh syndrome: with complex I deficiency – mutations in *NDUFS1, NDUFS4, NDUFS7, NDUFS8, NDUFV1*; with complex II deficiency – mutations in *SDHA*
Cardiomyopathy and encephalopathy with complex I deficiency – mutations in *NDFUS2*
Optic atrophy and ataxia with complex II deficiency–mutations in *SDHA*
Hypokalaemia and lactic acidosis with complex III deficiency – mutations in *UQCRB*

*Mutations involving assembly factors of the mitochondrial respiratory chain*
Leigh syndrome–mutations in *SURF* I and *LRPPRC*
Hepatopathy and ketoacidosis – mutations in *SCO1*
Cardiomyopathy and encephalopathy – mutations in *SCO2*
Leukodystrophy and renal tubulopathy – mutations in *COX10*
Hypertrophic cardiomyopathy – mutations in *COX15*
Encephalopathy, liver failure, and renal tubulopathy with complex III deficiency – mutations in *BCS1L*
Encephalopathy with complex V deficiency – mutations in *ATP12*

*Nuclear genetic disorders of intra-mitochondrial protein synthesis*
Leigh syndrome, liver failure, and lactic acidosis – mutations in *EFG1*
Lactic acidosis, developmental failure, and dysmorphism – mutations in *MRPS16*
Myopathy and sideroblastic anaemia – mutations in *PUS1*
Leukodystrophy and polymicrogyria – mutations in *EFTu*
Encephalomyopathy and hypertrophic cardiomyopathy – mutations in *EFTs*
Oedema, hypotonia, cardiomyopathy, and tubulopathy–mutations in *MRPS22*
Hypotonia, renal tubulopathy, and lactic acidosis – mutations in *RRM2B*

*Nuclear genetic disorders of mitochondrial protein import*
Mohr–Tranebjaerg syndrome or deafness-dystonia-optic neuronopathy (DDON) syndrome – mutations in *TIMM8A* (*DDP*)
Early-onset dilated cardiomyopathy with ataxia (DCMA) or 3-methylglutaconic aciduria, type V–mutations in *DNAJC19*

*Nuclear genetic disorders of mitochondrial DNA maintenance*
Chronic progressive external ophthalmoplegia – mutations in *POLG1, POLG2, PEO1, SLC25A4, RRM2B*, and *OPA1*)
Mitochondrial neurogastrointestinal encephalomyopathy – mutations in *TYMP*
Alpers syndrome–mutations in *POLG1* and *MPV17*
Infantile myopathy and spinal muscular atrophy – mutations in *TK2*
Encephalomyopathy and liver failure – mutations in *DGUOK*
Hypotonia, movement disorder and/or Leigh syndrome with methylmalonic aciduria – mutations in *SUCLA2* and *SUCLG1*
Optic atrophy, deafness, chronic progressive external ophthalmoplegia, myopathy, ataxia, and peripheral neuropathy – mutations in *OPA1*

*Miscellaneous*
Co-enzyme Q10 deficiency – mutations in *PDSS2*, *APTX*, *COQ2*, and *ETFDH*
Barth syndrome –mutations in *TAZ*
Cardiomyopathy and lactic acidosis associated with mitochondrial phosphate carrier deficiency – mutations in *SLC25A3*

Alpers syndrome: epilepsy, cortical blindness, micronodular hepatic cirrhosis, episodic psychomotor regression; Barth syndrome: cardiomyopathy, hypotonia, weakness, and neutropenia.

Nuclear mitochondrial disorders represent an important group of human diseases. They often pose significant diagnostic challenges related to their genetic and phenotypic heterogeneity, but they are increasingly being recognised, helped by greater clinical awareness and easier access to molecular genetic testing. A common feature shared by all these disorders is impaired mtDNA maintenance, which can lead to a reduction in mtDNA copy number, the accumulation of high levels of somatic mtDNA mutations, or both (Alberio et al., 2007; Chinnery and Zeviani, 2008; Spinazzola and Zeviani, 2009). The identification of these quantitative and qualitative mtDNA abnormalities in diagnostic specimens is therefore a key finding, suggesting an underlying nuclear defect, and helping to direct appropriate molecular investigations. MtDNA depletion is the pathological hallmark of several early-onset mitochondrial syndromes, and the clinical prognosis is often poor, due to the marked bioenergetic crisis caused by such a gross reduction in mtDNA copy number (Spinazzola et al., 2009). Interestingly, the observed mtDNA depletion can be highly tissue-specific, which partly explains the variability in disease presentation and severity.

**Fig. 4:**
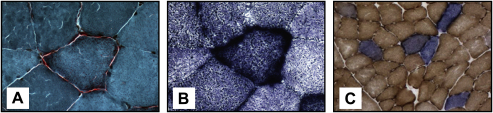
Skeletal muscle sections illustrating the characteristic histochemical features of mitochondrial dysfunction: (A) Ragged-red muscle fibre identified using the modified Gomori trichome stain. The red component of the staining mixture is selectively sequestered by mitochondria, which have accumulated in the subsarcolemmal region, giving the fibre an irregular red outline, (B) Serial section of the same muscle fibre after SDH staining. This is a more specific assay for detecting the subsarcolemmal accumulation of mitochondria, SDH being a specific marker for complex II activity, (C) Abnormal COX–SDH histochemistry from a patient with chronic progressive external ophthalmoplegia (CPEO) due to a single 5 kb mtDNA deletion, showing normal COX-positive (Brown) and energy deficient, COX-negative (Blue) muscle fibres.

A mosaic pattern of cytochrome *c* oxidase (COX) deficient fibres is frequently observed in muscle biopsies of patients with both primary mtDNA and nuclear mitochondrial disorders, with some of these fibres exhibiting abnormal accumulation of mitochondria in the subsarcolemmal space, giving the classical appearance of “ragged-red fibres” (RRFs) (Fig. 4). For nuclear genetic defects involving *POLG1* (Horvath et al., 2006; Tzoulis et al., 2006), *POLG2* (Longley et al., 2006), *PEO1* (Spelbrink et al., 2001), *SLC25A4* (Kaukonen et al., 2000), *TYMP* (Nishino et al., 1999), and more recently *OPA1* (Amati-Bonneau et al., 2008; Hudson et al., 2008; Stewart et al., 2008), the COX-defect is secondary to the accumulation of multiple mtDNA deletions, which have clonally expanded within individual cells to reach suprathreshold levels >70%. These deleted mtDNA species can be detected in homogenate DNA samples with Southern blot and long-range polymerase chain reaction (PCR), or more accurately quantified at the single-fibre level using real-time PCR assays (He et al., 2002; Taylor et al., 2004; Bua et al., 2006). As most mtDNA deletions involve critical tRNA and protein-encoding genes, OXPHOS is adversely affected, and this eventually leads to apoptotic cell loss and tissue dysfunction. Mutations in *POLG1* and *TYMP* have also been linked with the accumulation of somatic mtDNA point mutations (Del Bo et al., 2003; Nishigaki et al., 2003; Wanrooij et al., 2004). Although still speculative and controversial, these point mutations could compromise the replication machinery located within the D-Loop, thereby contributing to the formation of mtDNA deletions. It is fascinating that different mutations within the same gene can result in such a varied spectrum of secondary mtDNA abnormalities. The clarification of the secondary factors which dictate whether depletion, deletions, or point mutations predominate will provide crucial insights into the underlying disease mechanisms in nuclear mitochondrial disorders (Chinnery and Zeviani, 2008).

## 3 Leber hereditary optic neuropathy

### 3.1 Epidemiology

**Fig. 5:**
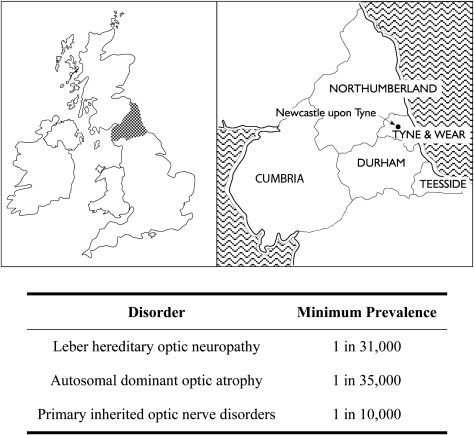
The minimum prevalence of inherited optic nerve disorders in the North of England.

The North of England has been relatively stable in terms of migratory flux, with a population of about three million, predominantly white, inhabitants (Fig. 5). As a result of the nature of healthcare provision in this region, over the past 20 years, patients with unexplained visual failure and suspected inherited optic neuropathies have been referred to the neuro-ophthalmology (PYWM and PGG) and neurogenetics (PFC) services in Newcastle upon Tyne for further assessment. This centralised referral pattern, in addition to active contact tracing, allowed us to determine for the first time the prevalence of LHON in a defined geographical region (Man et al., 2003). We found a minimum estimate of 1 in 31,000, and fairly comparable prevalence figures of 1 in 39,000 and 1 in 50,000 have since been reported in Dutch and Finnish population studies, respectively (Spruijt et al., 2006; Puomila et al., 2007). In Australia, the cause of visual impairment for about 2% of individuals on the national blind registry was optic atrophy secondary to LHON (Mackey and Buttery, 1992).

### 3.2 Primary mitochondrial DNA mutations

The majority of patients with LHON (90–95%) harbour one of three primary mtDNA point mutations: m.3460G>A (Howell et al., 1991a; Huoponen et al., 1991), m.11778G>A (Wallace et al., 1988), and m.14484T>C (Johns et al., 1992; Mackey and Howell, 1992). The m.11778G>A mutation was identified in 1988 by Wallace et al. (1988), and it is of special historical significance, being the first mtDNA substitution confirmed to cause human disease. The most prevalent LHON mutation in Northern Europe, Australia, and the Far East is m.11778G>A (Mackey et al., 1996; Mashima et al., 1998; Yen et al., 2002), but as a result of a founder event, m.14484T>C is the most common mutation (87%) among French Canadians. A number of other pathogenic mtDNA LHON variants have since been reported (Table 2), with some still awaiting full confirmation for pathogenicity, having been identified in only single families (Taylor et al., 2003). The distribution of these rarer mtDNA defects is not uniform, and the *MTND1* and *MTND6* gene regions are thought to be “mutational hotspots”, harbouring other LHON-causing mutations, in addition to m.3460G>A and m.14484T>C (Chinnery et al., 2001b; Valentino et al., 2004; Fraser et al., 2010). On close questioning, up to 60% of affected individuals report other family members with a pattern of early-onset visual failure, and a detailed family history should always be sought in suspected inherited optic neuropathy cases. *De novo* m.3460G>A and m.14484T>C mutations have been reported in LHON, but these are rare (Biousse et al., 1997; Man et al., 2003). Most presumed singleton cases are therefore probably due to difficulties in tracing back a more extensive family history.

As a follow-on to our original epidemiological study, about 3000 umbilical cord blood samples from a local birth cohort in the North of England were screened for specific mtDNA point mutations (Elliott et al., 2008). Nine healthy neonates were found to harbour one of the three primary LHON mutations, about 1 in every 350 births. In six of these neonates, the mutation was heteroplasmic, and of these cases, four were present at mutational levels less than 70%. There is clearly a large pool of these primary LHON mutations in the general population, and for heteroplasmic variants, the “mitochondrial bottleneck” will introduce shift in mitochondrial allele frequencies among future generations, directly influencing the risk of disease expression. The “mitochondrial bottleneck” is thought to be a protective mechanism which allows the rapid removal of deleterious mtDNA mutations from the genetic pool (Khrapko, 2008; Cree et al., 2009). The fertilised oocyte contains over 100,000 mtDNA molecules and during the early stages of development, there is a dramatic reduction in mtDNA copy number, down to 200–2000 copies before mtDNA replication is re-initiated. This decrease in the number of mitochondrial genomes repopulating the offspring of the next generation causes a sampling effect and accounts for the rapid changes in heteroplasmy levels. A pathogenic mtDNA variant would either be lost during transmission to the next generation, or it would quickly reach suprathreshold levels within an oocyte, increasing the likelihood of developmental arrest and its elimination. Even if a mature oocyte carrying a high proportion of the mutant species is successfully fertilised and a live birth results, there is a high probability that the affected individual’s fertility will be subnormal, which again serves to limit the transmission of mtDNA mutations. This situation clearly does not apply to LHON, as there is no evidence that mutational carriers have reduced fertility, and the majority of individuals, both affected and unaffected, are homoplasmic for the mtDNA mutation (Section 3.4.1).

### 3.3 Clinical manifestations

#### 3.3.1 Pre-symptomatic phase

In some asymptomatic LHON carriers, fundal abnormalities such as telangiectatic vessels around the optic discs, and fluctuating levels of retinal nerve fibre layer oedema have previously been observed (Nikoskelainen et al., 1996; Savini et al., 2005; Quiros et al., 2006). More detailed psychophysical testing can also uncover more subtle features of optic nerve dysfunction in some individuals, with loss of colour discrimination along the red–green axis, minimal central visual field changes on automated static perimetry, reduced contrast sensitivity, and subnormal visual electrophysiology (Salomao et al., 2004; Sadun et al., 2006; Sacai et al., 2010).

#### 3.3.2 Acute phase

Disease onset among LHON carriers is characterised by acute, painless loss of central vision, which is bilateral in about 25% of cases (Johns et al., 1993a; Nikoskelainen, 1994; Harding et al., 1995a; Nikoskelainen et al., 1996). If unilateral, the fellow eye is usually affected within six to eight weeks. There are rare cases of unilateral optic neuropathy (Nikoskelainen et al., 1996; Sugisaka et al., 2007), but these are the exceptions, second-eye involvement in LHON occurring invariably within 1 year of disease onset. The majority of carriers become symptomatic in the second and third decades of life, and over 90% of carriers who will experience visual failure will do so before the age of 50 years (Man et al., 2003; Spruijt et al., 2006). However, visual deterioration can occur anytime during the first to the seventh decade of life and LHON should be part of the differential diagnosis for all cases of bilateral, simultaneous or sequential optic neuropathy, irrespective of age, and especially in male patients (Shah et al., 2008; Yu-Wai-Man et al., 2008; Decanini-Mancera et al., 2009; Giraudet et al., 2010). Although one report identified females harbouring the m.11778G>A mutation as having a slightly increased age of onset compared to other groups (Harding et al., 1995b), gender and mutational status are not thought to significantly influence the timing or initial severity of visual loss.

Visual loss worsens over a period of four to six week, and it is severe, dropping to levels of 6/60 or worse, with a dense central or centrocaecal scotoma, and marked impairment in colour vision. Importantly, the pupillary light reflexes are thought to be relatively preserved in affected LHON patients compared with the extent of visual loss, and this can be a useful clinical sign (Wakakura and Yokoe, 1995; Kawasaki et al., 2010). In the acute stage, dilated fundal examination can be particularly informative, classical LHON cases exhibiting several distinct abnormalities such as vascular tortuosity of the central retinal vessels, swelling of the retinal nerve fibre layer, and a circumpapillary telangiectatic microangiopathy (Fraser et al., 2010). However, it must be stressed that in about 20% of LHON cases, the optic disc looks entirely normal, and these patients are sometimes labelled as having functional visual loss (Nikoskelainen et al., 1977; Harding et al., 1995a; Nikoskelainen et al., 1996).

#### 3.3.3 Chronic phase

Within six weeks, optic nerve pallor becomes apparent, initially more marked temporally due to early axonal loss within the papillomacular bundle. Pathological cupping of the optic disc can occur with more extensive loss of RGC axons, and it is not an uncommon finding in longstanding LHON cases. If a patient is first assessed at this late stage, it can be difficult to exclude compressive, infiltrative or inflammatory causes of a bilateral optic neuropathy, especially when there is no convincing maternal history of early-onset visual failure. The results of molecular genetic testing in some diagnostic laboratories can take up to 2 months, and in these cases, the appropriate investigations, including neuroimaging of the anterior visual pathways, should not be delayed in order to exclude the possibility of reversible causes.

#### 3.3.4 Visual prognosis

LHON causes significant visual impairment and in the majority of cases, visual recovery is minimal, the patient remaining within the legal requirement for blind registration. In the first year following disease onset, visual fields can improve with the appearance of small islands of vision (Mackey and Howell, 1992; Stone et al., 1992; Nikoskelainen et al., 1996). These fenestrations can help with scanning vision, especially if the central scotoma becomes concurrently less dense. The likelihood of visual recovery is greatest with the m.14484T>C mutation, and least with the m.11778G>A mutation, the m.3460G>A mutation having an intermediate visual prognosis (Harding et al., 1995a; Yu-Wai-Man et al., 2009b; Fraser et al., 2010). To objectively document the level of visual handicap experienced by LHON patients, we used the well-validated Visual Function Index (VF-14) questionnaire in a large cohort of 125 LHON pedigrees (Kirkman et al., 2009a). LHON had a negative detrimental impact on most activities of daily living, and quality of life, as assessed by the overall VF-14 score, was the worst compared with other acquired and inherited ophthalmological disorders.

#### 3.3.5 Extra-ocular LHON features

Although visual failure is the cardinal clinical feature, cardiac arrhythmias and neurological abnormalities such as peripheral neuropathy, myopathy, dystonia, and myoclonus have been reported to be more common among LHON carriers compared to controls (Bower et al., 1992; Nikoskelainen et al., 1994; Meire et al., 1995; Nikoskelainen et al., 1995; Mashima et al., 1996; McFarland et al., 2007; La Morgia et al., 2008). In a small number of families from Holland, Australia and North America, the reported extra-ocular features were particularly severe, with variable combinations of psychiatric disturbances, spastic dystonia, ataxia, and juvenile onset encephalopathy complicating the optic neuropathy. The phenotypic severity of these so-called “LHON plus” syndromes has been linked with specific mtDNA variants at m.4160T>C (Howell et al., 1991b), m.11696A>G and/or m.14596T>A (De Vries et al., 1996), and m.14459G>A (Jun et al., 1994; Gropman et al., 2004; Tarnopolsky et al., 2004). Two mtDNA point mutations affecting complex I activity, m.3376G>A and m.3697G>A, have also been identified in individuals with overlap clinical features of LHON and mitochondrial encephalomyopathy, lactic acidosis, and stroke-like episodes (MELAS) (Blakely et al., 2005; Spruijt et al., 2007).

Harding et al. (1992) originally described an intriguing association between the m.11778G>A primary mutation among female LHON carriers and demyelination. Following the onset of visual loss, these patients developed clinical and neuroimaging features indistinguishable from multiple sclerosis (MS), with characteristic periventricular white matter lesions on magnetic resonance imaging (MRI), and unmatched oligoclonal bands in their cerebrospinal fluid (Kellar-Wood et al., 1994; Jansen et al., 1996; Vanopdenbosch et al., 2000). Since this first description of Harding’s disease, further evidence has emerged in LHON and other mitochondrial disorders, which suggest that this association is unlikely to be a chance occurrence (Kovacs et al., 2005; Jaros et al., 2007; Carelli and Bellan, 2008; Verny et al., 2008; Yu-Wai-Man et al., 2010b). LHON female carriers are twice more likely to develop an MS-like illness compared with male carriers, and although there is a preponderance for the m.11778G>A mutation, this phenotype has also been observed with the m.3460G>A and m.14484T>C primary mutations (Sapey et al., 2001). More robust confirmatory epidemiological studies are required, but demyelination has been estimated to affect up to 1 in 20 LHON carriers (Palace, 2009), which is fifty times higher than the prevalence of MS in the general population (Fox et al., 2004; Koch-Henriksen and Sorensen, 2010). It has not yet been determined whether subclinical white matter MRI changes are present in asymptomatic LHON carriers or those who only manifest pure optic nerve involvement. If present, these again would suggest more widespread central nervous involvement in LHON, which becomes clinically manifest in only a subgroup of at-risk individuals (Inglese et al., 2001). Interestingly, the proton magnetic resonance spectroscopic (^1^H-MRS) profile of both affected and unaffected LHON carriers were found to be abnormal compared to healthy controls, with reduced creatine (Cr) and N-acetylaspartate (NAA) levels in normal-appearing white matter regions, suggesting an underlying mitochondrial metabolic deficit (Ostojic et al., 2009). A number of pathophysiological mechanisms have been put forward linking RGC loss, oligodendrocyte survival, and mitochondrial dysfunction in patients with LHON and MS-like features (Section 8).

### 3.4 Incomplete penetrance and gender bias

Two key features of LHON still remain unexplained; the marked incomplete penetrance and the significant gender bias in disease predisposition, with only 50% of male and 10% of female carriers eventually losing vision in their lifetime. The primary LHON mutation is a prerequisite, but secondary factors are clearly modulating the risk of visual loss. Their identification has proven challenging, and the accumulated evidence favours a complex disease model, with both genetic and environmental factors interacting to precipitate optic nerve dysfunction (Carelli et al., 2007b; Yu-Wai-Man et al., 2009b; Tonska et al., 2010).

#### 3.4.1 Mitochondrial genetic factors

Among heteroplasmic LHON carriers, visual loss only occurs if the mutational load exceeds 60%, the threshold required for triggering a bioenergetic defect (Chinnery et al., 2001a). However, incomplete penetrance is still observed among heteroplasmic carriers harbouring suprathreshold mutational levels, and over 80% of all LHON pedigrees are homoplasmic for the primary mtDNA mutation (Smith et al., 1993; Harding et al., 1995b; Man et al., 2003). Another possible mitochondrial modulating factor is the haplogroup background on which the LHON mutation is segregating. In a meta-analysis of 159 Caucasian LHON pedigrees, there was a significantly increased risk of visual failure when the m.11778G>A and m.14484T>C mutations occurred on a haplogroup J background, whereas m.3460G>A carriers were more likely to experience visual loss if they belonged to haplogroup K (Hudson et al., 2007b). A protective effect was conferred by haplogroup H, but only among m.11778G>A mutational carriers. The mitochondrial background also influenced the clinical expression of the m.11778G>A mutation among mainland Chinese LHON carriers, with haplogroup M7b1’2 increasing the risk of disease conversion, and haplogroup M8a having a protective effect (Ji et al., 2008). MtDNA haplogroups are defined by combinations of various polymorphic substitutions within the mitochondrial genome (Section 2.5). Some of these are non-synonymous, and they result in amino acid changes within mitochondrially-encoded subunits of the respiratory chain. Although it is convenient to view them as separate entities (Fig. 1), mitochondrial respiratory chain complexes do not exist in isolation, but they interact closely with one another forming so-called supercomplexes (Dudkina et al., 2010). Although speculative, these amino acid changes could induce subtle conformational changes, which affect the assembly and stability of these putative supercomplexes (Dudkina et al., 2005; Carelli et al., 2006; Hudson et al., 2007b; Pello et al., 2008). In support of this hypothesis, cybrid cell lines harbouring the m.11778G>A mutation had a lower oxygen consumption and a longer doubling time on a haplogroup J background, compared with other mtDNA haplogroups (Vergani et al., 1995). However, the link between specific mtDNA haplogroups and the risk of visual failure in LHON is not entirely clear-cut. A study of South-East Asian m.11778G>A LHON pedigrees found no significant association between specific mtDNA polymorphisms and the risk of developing overt optic nerve dysfunction (Tharaphan et al., 2006). Similarly, using *in vivo* ^31^P-MRS measurements, haplogroup J did not induce a more pronounced mitochondrial biochemical defect in the brain and skeletal muscle of affected m.11778G>A mutational carriers (Lodi et al., 2000).

#### 3.4.2 Nuclear genetic factors

**Fig. 6:**
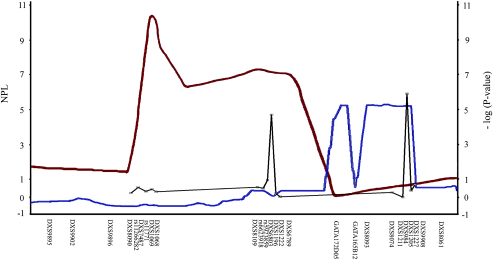
Summary of linkage studies investigating the existence of putative LHON nuclear modifiers on the X-chromosome. A nonparametric LOD score (NPL) >2 is indicative of significant linkage, and these chromosomal areas possibly harbour susceptibility loci which influence the risk of visual loss among LHON carriers. The different studies are colour coded: red (Hudson et al., 2005), blue (Shankar et al., 2008), and black (Ji et al., 2010). Reproduced with permission from Ji et al. (2010).

**Fig. 7:**
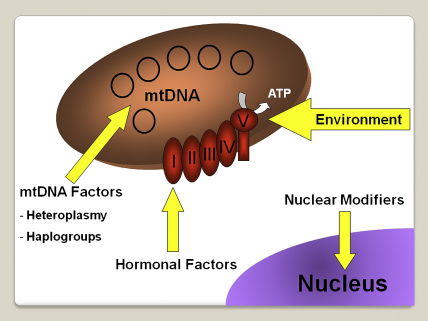
The complex interaction of genetic, hormonal, and environmental factors in the pathophysiology of LHON.

The marked male bias seen in LHON cannot be explained by mitochondrial genetic factors. Based on an extensive analysis of 31 large pedigrees totalling more than 1200 individuals, Bu and Rotter (1991, 1992) have proposed a two-locus model for visual failure in LHON. The segregation pattern was consistent with a visual-loss susceptibility gene on the X-chromosome, acting in synergy with the primary mtDNA mutation to precipitate visual loss among at-risk carriers. Male carriers have only one X-chromosome, and unlike female carriers, they cannot compensate for the inheritance of a putative X-linked visual-loss susceptibility allele (Oostra et al., 1996; Pegoraro et al., 1996; Hudson et al., 2007a). Three studies using microsatellite markers have now confirmed significant linkage on the X-chromosome, with some of these candidate regions showing areas of overlap (Figs. 6 and 7) (Hudson et al., 2005; Shankar et al., 2008; Ji et al., 2010). The actual gene or genes involved have yet to be identified, and more sophisticated bioinformatic tools are currently being applied for candidate gene analysis and to narrow down specific areas of interest. LHON could be an even more complex disorder than originally considered and the existence of autosomal nuclear modifiers remains a distinct possibility. A recent genome-wide study of nine large m.11778G>A Thai pedigrees found evidence of significant linkage on areas of chromosomes 3, 12, 13, and 18. Candidate gene regions were analysed with a tagging single nucleotide polymorphism (SNP) methodology, and two SNPs, rs3749446 and rs1402000, located within *PARL* (Presenilin-associated rhomboid-like) were associated with a statistically increased risk of phenotypic expression among LHON carriers (Phasukkijwatana et al., 2010). However, the association between these two *PARL* SNPs and visual loss was not replicated in an independent cohort of Chinese m.11778G>A LHON pedigrees (Zhang et al., 2010).

#### 3.4.3 Hormonal factors

Although much attention has been focused on possible secondary genetic modifiers in LHON, hormonal factors could also influence phenotypic expression. This hypothesis has recently been investigated by Giordano et al. (2010) using osteosarcoma-derived cybrid cell lines harbouring one of the three primary LHON mutations: m.3460G>A, m.11778G>A, and m.14484T>C. These mutant cybrids exhibited elevated ROS levels, decreased mitochondrial membrane potential, increased rates of apoptosis, and hyper-fragmented mitochondrial networks compared with controls. Interestingly, treatment with 17β-oestradiol had a mitigating effect on these pathological features. In addition, supplementation of these LHON cybrids with 17β-oestradiol led to increased cellular levels of the anti-oxidant enzyme superoxide dismutase (SOD) and to more efficient mitochondrial biogenesis. These results are very interesting, providing another explanation for the protective effect of female gender on LHON penetrance, and supporting the possible therapeutic use of oestrogen-like compounds in this disorder.

#### 3.4.4 Environmental factors

Two pairs of discordant monozygotic twins have been described, where one sibling has remained visually unaffected on long-term follow-up (Johns et al., 1993b; Biousse et al., 1997). These rare observations support an environmental component to the pathophysiology of optic nerve dysfunction in LHON, and there is increasing evidence in the literature supporting this hypothesis. The role of smoking and alcohol in LHON has been studied in a number of relatively small case-control studies, with contradictory findings (Chalmers and Harding, 1996; Tsao et al., 1999; Sadun et al., 2003; Newman, 2009). In one study, which included affected and unaffected siblings from 80 LHON sibships, high alcohol and tobacco consumption were not linked with an increased likelihood of visual failure (Kerrison et al., 2000). To further clarify this important issue, we conducted a multi-centre study of potential triggers in LHON, comparing the environmental exposure between 196 affected and 206 unaffected carriers (Kirkman et al., 2009b). Smoking was strongly associated with an increased risk of visual loss, and interestingly, there was a dose-response relationship, with the risk of visual loss being much greater in heavy smokers compared to light smokers. There was also a trend towards an increased risk of visual failure among heavy drinkers, but this effect was not as strong as smoking. Based on these results, LHON carriers should be strongly advised not to smoke and to moderate their alcohol intake, especially avoiding binge drinking episodes. Although no functional studies were performed, smoking could further impair mitochondrial OXPHOS, either through a direct effect on complex I activity, or by reducing arterial oxygen concentration (Gvozdjak et al., 1987; Vanjaarsveld et al., 1992; Yang et al., 2007; Kirkman et al., 2009b; Newman, 2009). Several other environmental triggers have been reported in LHON, including head trauma, acute physical illness, psychological stress, occupational exposure to chemical toxins such as 2,5-hexanedione, antiretroviral drugs, and anti-tuberculous agents (Sadun et al., 2003; Sanchez et al., 2006; Carelli et al., 2007a; Hudson et al., 2007b; Kirkman et al., 2009b). Most of these reports are clinical descriptions and they do not provide conclusive evidence for a causal relationship. However, Ghelli et al. (2009) have shown that 2,5-hexanedione had a mitochondrial toxic effect on LHON cybrids harbouring the m.11778G>A and m.14484T>C primary mutations. Of particular interest, an increased sensitivity to undergo apoptosis was noted on the haplogroup J mtDNA background, further highlighting the possible synergistic interactions between environmental and genetic risk factors.

### 3.5 Biochemical defect in LHON

All the three primary LHON mutations involve complex I subunits and their impact on mitochondrial OXPHOS has been extensively investigated using a wide range of cell types and biochemical assays. These *in vitro* studies have not always been consistent regarding the extent of the respiratory chain defect in LHON, but the overall evidence supports a predominant impairment in complex I-driven ATP synthesis (Table 3). *In vivo* ^31^P-MRS studies have also confirmed an underlying biochemical defect in LHON, with the m.11778G>A mutation resulting in the most pronounced reduction in mitochondrial ATP synthesis, followed by m.14484T>C, and m.3460G>A (Barbiroli et al., 1995; Lodi et al., 1997, 2002). With the caveat that RGCs were not directly assessed, an interesting observation from all these studies is that no significant difference in biochemical profile has ever been clearly demonstrated between affected and unaffected LHON carriers.

Oxidative stress leads to free radical production and ROS levels were found to be significantly increased in transmitochondrial cybrids carrying one of the three primary LHON mutations (Wong et al., 2002; Carelli et al., 2004b; Baracca et al., 2005; Floreani et al., 2005). LHON patients also have reduced α-tocopherol/lipid ratios and elevated 8-hydroxy-2-deoxygaunosine levels in their blood leukocytes, both of these biomarkers being indicative of increased ROS production (Klivenyi et al., 2001; Yen et al., 2004). LHON cybrids have impaired EAAT1 (Excitatory amino acid transporter 1) activity, which is highly relevant to RGC survival, as these transporters are actively involved in the uptake of glutamate into Muller cells of the inner retina (Beretta et al., 2004). The glutamate transport defect could be partially reversed by supplementing these LHON cybrids with antioxidants, supporting the use of these compounds in RGC neuroprotection (Ghelli et al., 2008; Sala et al., 2008). Increased ROS and glutamate excitotoxicity are potent inducers of apoptotic cell death, and in LHON, this process is likely to be both Fas-dependent and caspase-independent (Ghelli et al., 2003; Zanna et al., 2003).

## 4 Autosomal-dominant optic atrophy

### 4.1 Epidemiology

DOA affects at least 1 in 35,000 individuals in the North of England, a figure which is comparable to LHON (Yu-Wai-Man et al., 2010a). Based on the Danish Registry for Hereditary Eye Diseases, the prevalence of this optic nerve disorder in Denmark has been estimated at 1 in 12,000 (Toomes et al., 2001), a higher figure which could reflect the methodological criteria used. This study was also performed in the pre-molecular era and patients were included based on a clinical diagnosis of DOA.

### 4.2 Clinical features

Visual loss in DOA is insidious, invariably starting in the first two decades of life, and with a mean age of onset of 6–10 years. Compared with LHON, it has a milder phenotype and up to 1 in 4 patients are visually asymptomatic, optic atrophy only being detected through contact tracing of an affected proband (Kline and Glaser, 1979; Hoyt, 1980). Mean visual acuities of 6/18–6/60 have been reported in various case series, with visual acuities ranging from 6/6 to the detection of hand movement (Kjer, 1959; Eliott et al., 1993; Votruba et al., 1998b; Puomila et al., 2005; Cohn et al., 2008). There is a marked inter- and intra-familial variability in the rate of disease progression, but a significant proportion of patients (50–75%) will experience further worsening of their visual function in later life (Cohn et al., 2008; Yu-Wai-Man et al., 2010a,c). Although the overall visual prognosis is better compared with LHON, DOA still causes significant visual impairment with half of all patients failing the driving standards and being registered legally blind (Kjer et al., 1996; Votruba et al., 1998a; Cohn et al., 2007).

Most patients with DOA have a generalised dyschromatopsia and only a minority (10%) has pure tritanopia, which used to be considered a pathognomonic feature of this optic nerve disorder (Berninger et al., 1991). Due to the primary involvement of the papillomacular bundle, the most common visual defects are central, centrocaecal, and paracentral scotomas, with sparing of the peripheral field (Votruba et al., 1998a; Yu-Wai-Man et al., 2010a,c). Both magnocellular and parvocellular pathways seem to be similarly affected in DOA. Similar to LHON, the retino-tectal fibres mediating the pupillary light reflex are relatively preserved, and patients do not normally exhibit an afferent pupillary defect (Bremner et al., 2001).

**Fig. 8:**
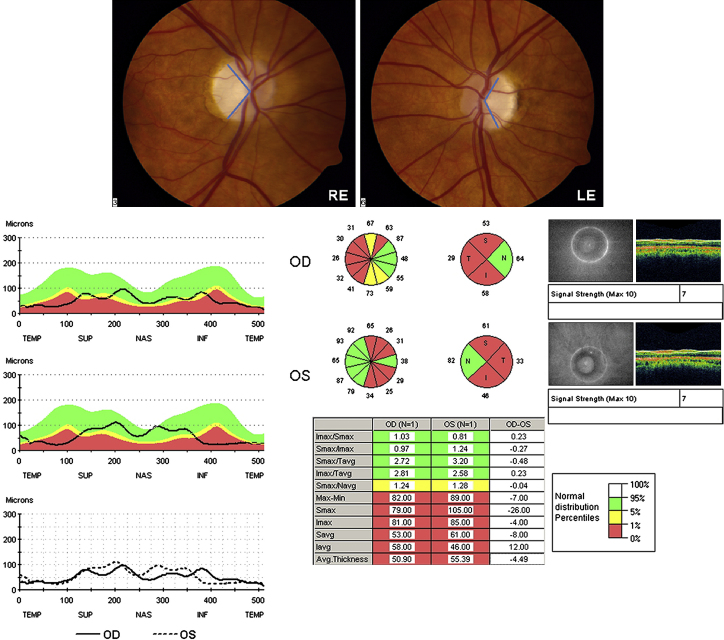
The optic disc appearance of a patient with a confirmed pathogenic *OPA1* mutation showing pallor of the neuro-retinal rim, which is more marked temporally. The bottom panels illustrate the pattern of retinal nerve fibre layer (RNFL) thinning seen in patients with *OPA1* mutations, with relative sparing of the nasal peripapillary quadrant. The RNFL profile for each eye is superimposed on the normal distribution percentiles, and compared with each other (Bottom left panel). Various measurement parameters are automatically generated by the analysis software including sectorial RNFL thickness for each individual quadrant and clock hour, and an overall value for the average RNFL thickness (Bottom middle panel). The normal distribution indices are colour-coded: (i) red <1%, (ii) yellow 1–5%, (iii) green 5–95%, and (iv) white >95% (Bottom right panel).

Optic disc pallor in DOA can be either diffuse, involving the entire neuro-retinal rim, or it can show a characteristic temporal wedge (Fig. 8). Pallor of the neuro-retinal rim can be subtle and in one report, nearly a third of all affected patients had normal looking optic discs on slit lamp biomicroscopy (Cohn et al., 2007). Based on our own experience, measurement of retinal nerve fibre layer (RNFL) thickness with optical coherence tomography (OCT) can be particularly helpful in equivocal cases, by confirming pathological RNFL thinning, especially in the temporal quadrant involving the papillomacular bundle (Ito et al., 2007; Kim and Hwang, 2007; Milea et al., 2010). Although not characteristic, other morphological disc features have been reported in DOA; saucerisation of the neuro-retinal rim, peripapillary atrophy, and enlarged cup-to-disc ratios greater than 0.5, the latter often leading to an erroneous diagnosis of normal tension glaucoma (Votruba et al., 1998a, 2003; Fournier et al., 2001; Yu-Wai-Man et al., 2010a,c).

### 4.3 Mutational spectrum

The majority (50–60%) of patients with DOA harbour mutations in the *OPA1* gene (OMIM 165500), and over 200 pathogenic mutations have been identified (e*OPA1* database at http://lbbma.univ-angers.fr/lbbma.php?id=9, Accessed 31st of August 2010) (Table 4). *OPA1* consists of 30 exons spread over 100 kb of genomic DNA and alternative splicing of exons 4, 4b and 5b results in eight different mRNA isoforms (Davies and Votruba, 2006; Olichon et al., 2006; Lenaers et al., 2009). *OPA1* mutations cluster in the GTPase domain (Exons 8–15) and dynamin central region (Exons 16–23), with single base-pair substitutions (69%) representing the most common mutational subtype, followed by deletions (26%), and insertions (5%) (Ferre et al., 2005, 2009). *OPA1* testing is usually performed with PCR-based sequencing protocols, and although cost and access are important practical issues, about 10–20% of patients who are found to be negative using these screening methods will harbour large-scale *OPA1* re-arrangements (Fuhrmann et al., 2009; Yu-Wai-Man et al., 2010a). The majority of *OPA1* mutations result in premature termination codons, with truncated mRNAs which are unstable and mostly degraded by protective cellular mechanisms (Pesch et al., 2001; Schimpf et al., 2006; Zanna et al., 2008). The reduction in OPA1 protein level is a major disease mechanism in DOA, and the importance of haploinsufficiency is further emphasised by rare families with microdeletions which span the entire *OPA1* coding region (Marchbank et al., 2002; Fuhrmann et al., 2009). However, about 30% of patients with DOA harbour missense *OPA1* mutations, and those located within the catalytic GTPase domain are more likely to exert a dominant-negative effect (Section 4.4) (Amati-Bonneau et al., 2008; Ferraris et al., 2008; Hudson et al., 2008; Yu-Wai-Man et al., 2010b).

Other genetic loci have been identified in families with DOA phenotypes (Table 4), but of these, only the causative gene for *OPA3* has been characterised. *OPA3* mutations were first identified in Iraqi Jewish families with Type III 3-methylglutaconic aciduria (Costeff syndrome); an autosomal recessive neurodegenerative disorder characterised by optic atrophy, progressive neurodegeneration, increased urinary levels of 3-methylglutaconic acid, and elevated plasma 3-methylglutaric acid levels (Costeff et al., 1989; Anikster et al., 2001). Pathogenic mutations were subsequently found in two independent French families segregating both optic atrophy and premature cataract in an autosomal-dominant mode of inheritance (ADOAC) (Reynier et al., 2004). However, *OPA3* mutations are likely to be extremely rare, especially in isolated optic atrophy cases. We did not identify any pathogenic *OPA3* variants in two large case series of *OPA1*-negative individuals, even with the use of a comparative genomic hybridization (CGH) assay to screen for large-scale *OPA3* re-arrangements (Yu-Wai-Man et al., 2010a,c).

### 4.4 Expanding clinical phenotypes

Even in the pre-molecular era, a number of pedigrees with DOA were described where optic neuropathy occurred in parallel with other clinical features such as sensorineural deafness and CPEO (Kjer, 1956, 1959; Kline and Glaser, 1979; Hoyt, 1980; Meire et al., 1985). *OPA1* mutations have been confirmed as the causative genetic defects in these syndromal forms of DOA, but until recently, these were thought to be rare manifestations among isolated families (Amati-Bonneau et al., 2003, 2005; Shimizu et al., 2003; Payne et al., 2004; Liguori, 2008). In our North of England inherited optic neuropathy cohort, DOA+ phenotypes were observed in 1 in 6 *OPA1* carriers, which clearly indicate that these syndromal variants affect a significant patient subgroup (Yu-Wai-Man et al., 2010a). We then conducted a multi-centre study of 104 patients manifesting DOA+ to define the phenotypic spectrum and natural history of these additional neurological complications (Yu-Wai-Man et al., 2010b). Bilateral sensorineural deafness beginning in late childhood and early adulthood was the most frequently observed extra-ocular manifestation, affecting nearly two-thirds of all cases. Other prominent clinical features then developed from the third decade of life onwards; ataxia, myopathy, peripheral neuropathy and CPEO. Unexpectedly, *OPA1* mutations were also identified in families previously labelled as having autosomal-dominant hereditary spastic paraplegia (HSP), and in individuals with visual failure complicated by a multiple sclerosis-like illness. Of note, there was a two- to three-fold increased risk of developing multi-system neurological disease with missense *OPA1* mutations located within the GTPase domain, suggesting deleterious gain-of-function mechanisms. Although these syndromal DOA+ variants show significant phenotypic variability even within the same family, a consistent finding is a worse visual prognosis among this patient subgroup. These observations are of major pathophysiological importance, highlighting the widespread deleterious consequences of *OPA1* mutations, not only for RGCs, but also for other neuronal populations, skeletal muscle, and extra-ocular muscle (Spinazzi et al., 2008; Zeviani, 2008; Mackey and Trounce, 2010). A key feature of these *OPA1* mutations is the induction of secondary mtDNA deletions, and their possible roles in triggering multi-system cellular dysfunction are currently being investigated (Yu-Wai-Man et al., 2010d).

### 4.5 OPA1 and OPA3 protein functions

OPA1 is highly expressed within the RGC layer, but it is a ubiquitous protein, and abundant levels have also been identified in photoreceptors, and other non-ocular tissues such as the inner ear and the brain (Aijaz et al., 2004; Pesch et al., 2004; Bette et al., 2005; Kamei et al., 2005; Akepati, 2008). OPA1 belongs to a large family of mechanoenzymes, which is characterised by a highly conserved, dynamin GTPase domain (Davies and Votruba, 2006; Lenaers et al., 2009). OPA1 is a transmembrane protein embedded within the mitochondrial inner membrane, and it mediates several interrelated cellular functions. One important aspect relates to its pro-fusion properties and unsurprisingly, the pathological hallmark of *OPA1* mutations is mitochondrial network fragmentation, the isolated mitochondria becoming dysmorphic, with aberrant balloon-like enlargements, disorganised cristae, and paracrystalline inclusion bodies (Kamei et al., 2005; Olichon et al., 2007; Amati-Bonneau et al., 2008; Chevrollier et al., 2008; Zanna et al., 2008). GTP hydrolysis activates membrane tubulation and this GTPase activity is thought to be enhanced following interaction of OPA1 with inner membrane phospholipids such as cardiolipin (Ban et al., 2010). Cytochrome *c* molecules are normally sequestered within the tight cristae junctions, and as they leach out into the cytosol, the apoptotic cascade is potentiated contributing to cell death and tissue dysfunction (Cipolat et al., 2006; Frezza et al., 2006; Suen et al., 2008). OPA1 also regulates OXPHOS by interacting directly with the respiratory chain complexes, controlling their delicate assembly, and facilitating the efficient shuttling of electrons between complexes (Zanna et al., 2008). Fibroblasts harbouring *OPA1* mutations have reduced mitochondrial membrane potentials and ATP synthesis was found to be significantly impaired (Amati-Bonneau et al., 2005; Chevrollier et al., 2008), secondary to a predominantly complex I defect (Zanna et al., 2008). With the use of *in vivo* phosphorus magnetic resonance spectroscopy (^31^P-MRS), these biochemical defects have also been demonstrated in the calf muscles of patients harbouring *OPA1* mutations (Lodi et al., 2004; Yu-Wai-Man et al., 2010f). Interestingly, it is now apparent that OPA1 plays a critical role in maintaining the integrity of the mitochondrial genome. High levels of COX-negative fibres have been identified in skeletal muscle biopsies from patients with DOA+ phenotypes, and this has been conclusively linked to the accumulation of multiple mtDNA deletions (Stewart et al., 2008; Yu-Wai-Man et al., 2010b,d).

OPA3 has a mitochondrial targeting domain, and it was thought to localise to the mitochondrial inner membrane (Anikster et al., 2001; Ho et al., 2008; Huizing et al., 2010). However, a recent study challenged this view, suggesting instead that OPA3 is an integral component of the mitochondrial outer membrane, with its C-terminus exposed to the cytosol (Ryu et al., 2010). Mitochondrial fragmentation was induced both with mutant forms of the protein, and following overexpression of the wild-type protein, indicating an important pro-fission role for OPA3 (Ryu et al., 2010). The cells studied had an increased sensitivity to various pro-apoptotic stimuli, a phenomenon which was also observed in fibroblasts collected from an affected patient belonging to one of the French ADOAC families (Reynier et al., 2004).

## 5 Other mitochondrial optic neuropathies

### 5.1 Charcot-Marie-Tooth disease

Charcot-Marie-Tooth (CMT) disease is a heterogeneous group of inherited peripheral neuropathies, and as a group they are one of the most common inherited human disorders, affecting at least 1 in 2500 individuals (Zuchner and Vance, 2006; Pareyson and Marchesi, 2009). Both motor and sensory nerves are affected resulting in distal limb weakness, sensory loss, decreased deep tendon reflexes, and foot deformities. A specific CMT subtype, hereditary motor and sensory neuropathy type VI (HMSN-VI, OMIM 601152), is caused by *MFN2* mutations (1p36.2) (Zuchner et al., 2004). In addition to early onset, severe peripheral neuropathy, affected individuals develop progressive optic nerve dysfunction starting in later childhood (Zuchner et al., 2006). Visual acuity usually deteriorates to levels of 6/60 or worse, but a subset of patients with HMSN-VI can experience sometimes dramatic visual recovery in later life. MFN1 and MFN2 are mitochondrial outer membrane proteins with dynamin GTPase domains, and they share a remarkable degree of structural and functional complementarity with OPA1 (Cartoni and Martinou, 2009; Chen and Chan, 2009). These three proteins interact closely with each other to coordinate the various steps involved in mitochondrial membrane fusion. MFN2 is also thought to exert a direct influence on mitochondrial biogenesis by regulating the expression of nuclear-encoded respiratory chain subunits (Pich et al., 2005). Consistent with this hypothesis, fibroblasts from patients harbouring *MFN2* mutations exhibit a mitochondrial coupling defect, with impaired membrane potential and reduced OXPHOS capacity (Loiseau et al., 2007).

### 5.2 Hereditary spastic paraplegia

The clinical hallmark of HSP is slowly progressive lower limb spasticity and weakness. Its prevalence has been estimated at 3–10 per 100,000 in Europe, and the age of onset varies according to the causative genetic defect (Salinas et al., 2008). HSP is classified into pure and complicated forms, depending on whether additional clinical features are present besides spastic paraplegia, such as optic atrophy, ataxia, peripheral neuropathy, extrapyramidal deficits, and cognitive decline (Harding, 1983). HSP is genetically heterogeneous with 41 mapped loci. So far, 17 genes have been identified, providing valuable insights into the various pathogenetic mechanisms which trigger axonal degeneration along the corticospinal tracts (Shy et al., 2002; Reid, 2003; Salinas et al., 2008).

OPA1 is cleaved by mitochondrial proteases following import into the mitochondrial intermembrane space, and these post-translational maturational steps are critical, generating long (L) and short (S) forms of the protein (Pellegrini and Scorrano, 2007; Lenaers et al., 2009; Martinelli and Rugarli, 2010). On their own, the L and S forms of OPA1 have little functional activity, but when co-expressed, they complement each other, triggering mitochondrial network fusion. One of these key mitochondrial proteases is paraplegin, which is encoded by *SPG7* (16q24.3). Mutations in *SPG7* have been identified in an autosomal recessive form of HSP, and in some patients, bilateral optic neuropathy is a prominent clinical feature further complicating the neurological phenotype (HSP-7, OMIM 607259) (Casari et al., 1998). Muscle biopsies from two severely affected individuals with HSP-7 showed classical histochemical changes of mitochondrial dysfunction with ragged-red fibres and COX-negative fibres. Transmission electron microscopy confirmed an accumulation of abnormal mitochondria containing paracrystalline inclusion bodies. Biochemical studies performed on cultured myoblasts harbouring *SPG7* mutations have revealed a reduction in citrate synthase-corrected complex I activity, again suggesting that impaired OXPHOS plays an important role in the pathogenesis of HSP, and by extension the optic neuropathy observed in a subgroup of patients (Wilkinson et al., 2004).

### 5.3 Friedreich ataxia

Friedreich ataxia (FRDA) is an autosomal recessive disorder caused by pathological GAA trinucleotide repeat expansions in the *FXN* gene (9q13-q21.1, OMIM 229300) (Campuzano et al., 1996). The encoded protein frataxin is directed to the mitochondrial inner membrane and it is involved in the assembly of iron–sulphur clusters, which are critical components of the mitochondrial respiratory chain complexes (Rouault and Tong, 2005; Stemmler et al., 2010). *FXN* mutations therefore impair OXPHOS and they also result in abnormal accumulation of intra-mitochondrial iron, which eventually reaches toxic levels (Rotig et al., 1997, 1998). Because frataxin has anti-oxidant properties, cellular defences against ROS are impaired in FRDA, and this likely further exacerbates neuronal loss (Rustin et al., 1999; Rotig et al., 2002; Schmucker and Puccio, 2010).

Patients with FRDA usually become symptomatic in the second decade of life, with progressive gait ataxia, loss of the deep tendon reflexes, dysarthria, distal limb weakness, pes cavus, scoliosis, and arrhythmias secondary to hypertrophic cardiomyopathy (Durr et al., 1996). In a recent study of 26 patients with genetically-confirmed FRDA, all patients had evidence of optic nerve dysfunction, although only five were visually symptomatic (Fortuna et al., 2009). The optic neuropathy differed from that observed in LHON and DOA, with a diffuse and progressive pattern of RNFL loss, and no preferential involvement of the papillomacular bundle. Interestingly, the pathological process in FRDA extended to the post-geniculate optic radiations, and the involvement of the anterior and posterior visual pathways seemed to proceed independently of each other. Altogether, these findings suggest that *FXN* mutations probably cause RGC loss via other disease mechanisms compared with *OPA1* and the primary mtDNA LHON mutations.

### 5.4 Autosomal recessive non-syndromal optic atrophy

Autosomal recessive optic neuropathies are rare and the visual phenotype is usually overshadowed by other more prominent neurodegenerative features. Using homozygosity mapping, Hanein et al. (2009) identified mutations in *TMEM126A* (11q14.1-q21, OMIM 612989) in a large, inbred, Algerian family segregating pure optic atrophy. TMEM126A is conserved in higher eukaryotes and it encodes a transmembrane mitochondrial protein, which is found at high levels within the RGC layer and optic nerve head. The exact localisation and function of this protein remain to be clarified, but *TMEM126A* mutations do not cause mitochondrial network fragmentation or mtDNA depletion. A novel nonsense *TMEM126A* mutation has since been reported where affected family members had subclinical auditory neuropathy in addition to progressive visual failure. The clinical phenotype associated with *TMEM126A* mutations could therefore expand further as more families with syndromal optic atrophy are screened for this particular gene (Meyer et al., 2010).

### 5.5 Mitochondrial protein-import disorders

Mohr–Tranebjaerg syndrome or deafness-dystonia-optic neuronopathy (DDON) syndrome is caused by loss-of-function mutations in the *TIMM8A* gene (Xq22, OMIM 300356) (Tranebjaerg et al., 1997). The clinical phenotype includes prelingual or postlingual sensorineural deafness, dystonia and ataxia in late childhood, visual failure with optic atrophy from the age of 20 years, and cognitive decline with psychiatric disturbances before the age of 50 years (Tranebjaerg et al., 1997; Tranebjaerg, 2003). Electrophysiological studies indicate that the visual loss in DDON is secondary to RGC loss, and the visual prognosis is poor, with most patients registered legally blind by the age of 40 years (Tranebjaerg et al., 1995, 2001; Ponjavic et al., 1996; Ujike et al., 2001). TIMM8A assembles as a 70 kDa hetero-oligomeric complex in the mitochondrial intermembrane space, and in conjunction with TIMM13, another mitochondrial membrane translocase, this complex facilitates the import and insertion of inner membrane proteins (MacKenzie and Payne, 2007). COX-negative fibres were not present in muscle biopsy specimens from affected patients and there was no alteration in mitochondrial network morphology (Binder et al., 2003; Blesa et al., 2007). However, there was evidence of an underlying mitochondrial biochemical defect, with reduced complex I–IV enzymatic activities.

Dilated cardiomyopathy with ataxia (DCMA) or 3-methylglutaconic aciduria, type V (OMIM 610198) is an autosomal recessive disorder found in the Dariusleut Hutterite population of Canada and the Northern United States. The clinical phenotype is characterised by growth failure, severe, early-onset cardiomyopathy, and a cerebellar syndrome with ataxia. Optic atrophy has been reported in some patients with DCMA, although it is not a cardinal feature of this syndrome (Davey et al., 2006). Homozygous mutations in the *DNAJC19* gene (3q26.33) have recently been identified in these inbred families, a G–C transversion within the splice acceptor site of intron 3 (Davey et al., 2006). The DNAJC19 protein localises to the mitochondrial compartment and although its subcellular localisation requires further investigation, it is thought to be the human orthologue of the yeast Tim14 protein (Mokranjac et al., 2006). Tim14 is an integral mitochondrial inner membrane protein and together with other mitochondrial translocases, it actively participates in protein import into the mitochondrial matrix compartment. Both DDON and DCMA are therefore due to defective mitochondrial protein-import systems, and it is fascinating that in both disorders optic nerve involvement is observed, albeit as part of a more widespread multi-systemic degeneration (MacKenzie and Payne, 2007).

### 5.6 Mitochondrial encephalomyopathies

Optic neuropathy can also develop in the following classical mitochondrial syndromes, although it is usually a secondary feature, overshadowed by other more prominent neurological and ocular manifestations: MELAS, myoclonic epilepsy and ragged-red fibres (MERRF), CPEO, KSS, maternally inherited Leigh syndrome (MILS), and mitochondrial neurogastrointestinal encephalomyopathy (MNGIE) (Gronlund et al., 2010).

### 5.7 Overlapping phenotypes

The concept of mitochondrial optic neuropathies has greatly expanded over the years and this trend is set to continue as new genes with mitochondrial-related functions are identified in other inherited optic nerve disorders. What is truly remarkable is the overlapping phenotypes seen with this group of disorders, for example among *OPA1* carriers, some patients develop neurological features indistinguishable from HSP, others develop a pattern of peripheral neuropathy with a similar disease course to CMT, and others still will develop a prominent cerebellar syndrome consistent with FRDA. These shared clinical features also apply to the optic nerve phenotype, as seen with LHON and DOA, the two most common inherited optic neuropathies diagnosed in the general population. As described in earlier sections, LHON and DOA have distinct clinical presentations which usually allow for their easy differentiation, but with the greater availability of genetic testing, it is now clear that a degree of phenotypic overlap exists. Although unusual, some patients with genetically-confirmed *OPA1* mutations have been described with acute and even reversible visual loss (Cornille et al., 2008; Nochez et al., 2009), and a subgroup of LHON patients can present with a slowly progressive optic neuropathy, more suggestive of DOA (Barboni et al., 2006).

## 6 Toxic optic neuropathies

### 6.1 Smoking, alcohol, and nutritional deprivation

Individuals with excessive alcohol and tobacco intake can develop a bilateral optic neuropathy, with slowly progressive visual loss, dyschromatopsia, and centrocaecal scotomas (Rizzo and Lessell, 1993; Lessell, 1998). This entity is sometimes referred to as “tobacco-alcohol amblyopia”, but there is persisting debate regarding its actual aetiology (Grzybowski, 2007; Grzybowski and Holder, 2010). It is possible that alcohol and tobacco *per se* are not the main or only causative factors, with other confounding variables such as nutritional deprivation contributing to the observed RGC-toxic effect. An epidemic of optic neuropathy was observed in Cuba in the early 1990s at a time of deteriorating socioeconomic conditions within this country (Sadun et al., 1994; Bern et al., 1995). This outbreak of optic neuropathy has been linked with chronic malnutrition combined with high levels of tobacco and cassava consumption. Cassava contains naturally-occurring cyanogenic glucosides and the occurrence of cyanide poisoning has been well described, especially during periods of famine. In terms of disease mechanisms, it is interesting that primary mtDNA LHON mutations have been detected in patients labelled as having “tobacco-alcohol amblyopia” (Cullom et al., 1993; Purohit and Tomsak, 1997). Moreover, smoking has recently been identified as a strong risk factor for visual loss among LHON mutational carriers, with heavy smokers having a higher risk than light smokers (Kirkman et al., 2009b). However, it should be noted that during the Cuban optic neuropathy outbreak, there was no evidence of increased disease penetrance in a large Cuban m.11778G>A family (Newman et al., 1994).

### 6.2 Chloramphenicol

With the advent of safe, broad spectrum antibiotics, systemic chloramphenicol is now rarely used for treating severe, disseminated infections. However, until the late 1970s, it was frequently used in the management of patients with cystic fibrosis, and a rare but serious complication noted in this group of patients was bilateral optic neuropathy (Godel et al., 1980; Yunis, 1989; Venegas-Francke et al., 2000). Post-mortem studies performed on patients who died shortly afterwards showed complete loss of RGCs within the distribution of the papillomacular bundle, with relative sparing of the nasal and peripheral retina (Cogan et al., 1973). There was no evidence of inflammation within the optic nerve but there was mild gliosis and demyelination in both the pre- and post-laminar segments. Transmission electron microscopy of haemopoetic cells collected from patients with chloramphenicol-induced bone marrow suppression revealed swollen mitochondria with disrupted cristae architecture, in addition to abnormal intra-mitochondrial iron deposits (Smith et al., 1970; Yunis and Smith, 1970; Skinnider and Ghadially, 1976). These findings strongly suggest that the pathological changes in chloramphenicol optic neuropathy are due to a similar mitochondrial toxic effect. Chloramphenicol is a powerful inhibitor of both bacterial and mitochondrial ribosomal protein synthesis, resulting in mitochondrial stress and a marked reduction in cellular ATP biosynthesis (Bhat et al., 1981; Li et al., 2010).

### 6.3 Linezolid

Linezolid is an oxazolidinone antibiotic that is increasingly being used to treat drug-resistant, gram positive organisms such as methicillin-resistant *Staphylococcus aureus* (MRSA) and vancomycin-resistant *Enterococcus* (VRE) (De Vriese et al., 2006). Overall, it is a safe antibiotic agent when used for up to 28 days, with only minor side effects reported in large Phase III clinical trials. However, about 20 cases of linezolid-associated optic neuropathy (LION) have been reported in the literature, and a common feature of this rare association seems to be an extended duration of treatment (5–50 months) (McKinley and Foroozan, 2005; Saijo et al., 2005; Rucker et al., 2006; Javaheri et al., 2007). Patients present with typical features of a bilateral optic neuropathy and at first presentation, visual acuities ranging from 6/9 to counting fingers have been reported. Early in the course of the disease, optic disc swelling and marked thickening of the RNFL have also been documented, the latter indicating axonal RGC swelling and strikingly reminiscent of the pathological process in acute LHON. Unlike linezolid-induced peripheral neuropathy which is irreversible, full visual recovery is possible following discontinutation of linezolid therapy. It is therefore crucial for clinicians to be aware of this possibly devastating complication among patients receiving linezolid treatment beyond the 28-day safe limit. Intriguingly, mitochondrial dysfunction seems to be central to the pathophysiology of LION, and a number of hypotheses have been proposed based upon the mechanism of action of linezolid. The antibacterial effect of linezolid is due to inhibition of bacterial protein synthesis, with the active moiety binding to the 23S rRNA of the 50S ribosomal subunit, thereby preventing the formation of the 70S RNA initiation complex (Javaheri et al., 2007). Mammalian ribosomes lack the 50S component and they are therefore not affected by linezolid. Although speculative, it is possible that mitochondrial ribosomes are susceptible to the sustained effect of long-term linezolid because of their closer evolutionary similarities to bacterial ribosomes. Consistent with this mechanism, mitochondrial respiratory chain activity was significantly depressed in a range of tissues; muscle, liver, and kidney, biopsied from a patient who developed optic neuropathy, encephalopathy, skeletal myopathy, lactic acidosis, and renal failure after a 4-month course of linezolid (De Vriese et al., 2006). This finding was further substantiated in an experimental rat model, with linezolid inducing a dose- and time-dependent decrease in the activity of complex I, and to a lesser extent that of complex IV (De Vriese et al., 2006).

### 6.4 Erythromycin

Luca et al. (2004) reported on a 23-year-old male who developed bilateral optic neuropathy following treatment with erythromycin. His visual acuity was severely reduced to counting fingers in both eyes and bilateral diffuse optic nerve pallor was observed, more marked temporally. A homoplasmic m.11778G>A mutation was identified, and to investigate a possible link between erythromycin and the onset of visual failure in this LHON carrier, cybrid osteosarcoma cells were established harbouring the patient’s mtDNA. When cultured in media containing erythromycin, a marked impairment in mitochondrial protein synthesis was noted and this was a dose-dependent effect. Similar to linezolid, erythromycin binds to the 23S rRNA molecule of the 50S ribosomal subunit blocking the exit of the growing polypeptide chain (Thomas and Wilkie, 1968; Tok and Bi, 2003). Erythromycin and, by extension, other macrolide antibiotics are therefore potential mitochondrial toxins and their use should be avoided in patients with mitochondrial genetic disorders, especially if other alternatives are available.

### 6.5 Ethambutol

Ethambutol is an important first-line drug used for treating tuberculosis and 2–6% of patients receiving this anti-mycobacterial drug will develop optic neuropathy, a serious side effect which is duration- and dose-dependent (Melamud et al., 2003; Lee et al., 2008). Optic nerve involvement is rare with treatment duration of less than 2 months, and although visual function can improve following discontinuation of the drug, in some cases, it is irreversible (Kim and Hwang, 2009). Several studies have confirmed a direct, toxic effect of ethambutol on RGC survival, and various pathophysiological mechanisms have been proposed including glutamate excitotoxicity, zinc-mediated lysosomal membrane permeabilisation, and disruption of mitochondrial complex IV activity through a copper-chelating action (Heng et al., 1999; Chung et al., 2009). Dotti et al. (1998) were the first to describe the onset of bilateral, subacute visual loss in a LHON m.11778G>A carrier, 8 months following the initiation of treatment for pulmonary tuberculosis. Since then, other case reports of ethambutol-induced optic neuropathy among LHON carriers have been reported, suggesting a synergistic deleterious effect of this anti-mycobacterial drug on the background of a pre-existing pathogenic mtDNA mutation (De Marinis, 2001; Hwang et al., 2003; Ikeda et al., 2006; Pradhan et al., 2010; Seo et al., 2010). Against this argument, the m.11778G>A mutation did not further enhance ethambutol cytotoxicity in NT2/D1 teratoma-derived cybrids, but this could simply reflect a tissue-specific effect, the latter being more pronounced in RGCs (Pommer et al., 2008).

The link between ethambutol therapy and inherited mitochondrial optic neuropathies was further highlighted by Guillet et al. (2010). They unexpectedly identified a pathogenic *OPA1* mutation in a male patient who developed bilateral, sudden, central visual loss 3 months after starting treatment for primary tuberculosis on a standard regimen of ehthambutol, isoniazid, and rifampicin. His visual acuity was severely affected, with caecocentral scotomas, and no visual recovery was noted following withdrawal of ethambutol. The acute presentation observed in this case is somewhat atypical for DOA and functional studies performed on cultured fibroblasts supported a role for ethambutol in precipitating an accelerated loss of RGCs. Compared with control fibroblasts similarly treated with ethambutol, a more marked biochemical uncoupling defect was observed, with loss of mitochondrial membrane potential, mitochondrial fragmentation, disturbed calcium homeostasis, and the accumulation of abnormal intracellular vacuoles (Guillet et al., 2010).

### 6.6 Antiretroviral drugs

There are suggestive reports of visual loss being triggered among LHON carriers following the initiation of highly active antiretroviral therapy (HAART) for newly diagnosed HIV infections (Shaikh et al., 2001; Mackey et al., 2003). These HIV patients were all male and harboured either the m.11778G>A or the m.14484T>C primary mtDNA mutations. The possible mechanisms have focused mainly on a particular class of antitretrovirals, the nucleoside analogue reverse transcriptase inhibitors (NRTIs), which can induce mitochondrial toxicity. NRTIs can inhibit mitochondrial polymerase gamma (POLG), a key component of the mtDNA replication machinery (Samuels, 2006; Scruggs and Naylor, 2008). A well reported side effect of NRTI therapy is mtDNA depletion, the resulting biochemical defect leading to lactic acidosis, hepatotoxicity, cardiomyopathy, generalised myopathy, and peripheral neuropathy (Cote et al., 2002; Frerichs et al., 2002; Maagaard et al., 2006). Fascinatingly, HAART has also been linked with the development of a CPEO phenotype in a limited number of HIV patients, with a gradual onset of ophthalmoplegia and ptosis (Pfeffer et al., 2009). One attractive hypothesis is that patients harbouring specific SNPs within *POLG* are at a higher risk of developing mitochondrial toxicity to NRTIs, with the HIV virus itself having an additive detrimental effect, further exacerbating mitochondrial dysfunction (Nolan and Mallal, 2004; Maagaard et al., 2006; Samuels, 2006). The mitochondrial protein profile of treatment-native HIV patients and those receiving HAART therapy have recently been investigated in blood leukocytes using a proteomic-based approach. Both groups of patients showed downregulated levels of prohibitins, important mitochondrial enzymes closely involved in OXPHOS and the maintenance of the mitochondrial network (Artal-Sanz and Tavernarakis, 2009; Ciccosanti et al., 2010). Additional studies are needed to determine whether fibroblasts from HIV patients, on or off HAART therapy, show any evidence of mitochondrial network fragmentation. Given the emerging links between HIV, HAART therapy, and mitochondrial dysfunction, it is not unreasonable to speculate that LHON carriers would be at an increased risk of visual loss in this particular context, and should therefore be adequately counselled should they require treatment with antiretrovirals.

## 7 Glaucoma

### 7.1 Normal tension glaucoma

Primary open angle glaucoma (POAG) affects over 60 million people worldwide and it is the second most common cause of legal blindness in developed countries (Bunce and Wormald, 2006; Quigley and Broman, 2006). RGCs are lost at an accelerated rate compared with normal ageing, resulting in pathological disc cupping and visual field defects. POAG is a complex disease and several genetic susceptibility loci have been identified in populations from different ethnic backgrounds (Libby et al., 2005; Wiggs, 2007; Ray and Mookherjee, 2009). The major risk factor for POAG is elevated intraocular pressure (IOP), but in about a third of patients, IOPs never exceed the statistical normal threshold of 21 mmHg (Boland and Quigley, 2007). Although lowering IOP can reduce visual field progression in normal tension glaucoma (NTG), additional IOP-independent risk factors are considered to be more important in the pathophysiology of NTG than in high tension glaucoma (HTG) (Alward et al., 1998; Schulzer et al., 1998; Cheng et al., 2009). The pattern of RGC loss in these two disease subgroups has been extensively investigated, and in most studies, NTG patients tended to have steeper, more focal field defects, which were located closer to fixation compared with HTG patients (Araie et al., 1997; Ahrlich et al., 2010). It is therefore not surprising that patients with DOA are often misdiagnosed as having NTG, especially if there is no family history of early-onset visual loss (Trobe et al., 1980; Fournier et al., 2001). Furthermore, disc pallor in DOA can be quite subtle, involving only the temporal segment, and about half of all patients have excavated discs, with cup-to-disc ratios greater than 0.5 (Votruba et al., 1998a, 2003). Some investigators have even argued, rather provocatively, that NTG is in actual fact an inherited optic neuropathy representing a *forme fruste* of DOA (Buono et al., 2002).

### 7.2 *OPA1* polymorphisms

*OPA1* was an obvious candidate disease-susceptibility gene in glaucoma and we recently carried out a systematic screen of the entire coding region using a well-characterised POAG cohort from the North of England (Yu-Wai-Man et al., 2010e). No pathogenic sequence variants were identified ruling out *OPA1* as a monogenic risk determinant for this disorder. Instead, we found a statistically significant association between two specific intronic *OPA1* SNPs, IVS8+4c>t and IVS8+32t>c, and the risk of developing NTG but not HTG. Three other studies have found a similar association between these two *OPA1* SNPs and NTG, supporting a possible role of the OPA1 protein in modulating disease susceptibility to glaucoma (Aung et al., 2002; Powell et al., 2003; Mabuchi et al., 2007). However, these findings have not been replicated in other populations, and functional studies are needed to clarify the mechanisms by which *OPA1* SNPs could exacerbate RGC loss and increase the risk of developing NTG (Sato et al., 2003; Woo et al., 2004; Kumaramanickavel et al., 2005; Yao et al., 2006; Wolf et al., 2009).

### 7.3 *OPTN* mutations

Mutations in four genes have been identified in families with POAG: *optineurin* (*OPTN*, OMIM 602432), *myocilin* (*MYOC*, OMIM 610652), *CYP1B1* (OMIM 601771), and *WDR36* (OMIM 609669). *OPTN* mutations are present in about 17% of families with autosomal-dominant NTG (Rezaie et al., 2002), but overall they are relatively rare, accounting for less than 2% of sporadic cases (Weisschuh et al., 2005; Hauser et al., 2006; Sripriya et al., 2006). These studies, including the original report of *OPTN* mutations in NTG, also suggest that the p.M98K *OPTN* variant could be an important disease risk factor, being present in 10–15% of glaucoma subjects compared with only 2–4% of normal controls. In one study, the p.M98K variant was not found to be associated with DOA and LHON, and it did not influence the severity or risk of visual loss (Craig et al., 2006).

Intriguingly, *OPTN* mutations have also recently been identified in Japanese individuals from consanguineous marriages and with a familial form of amyotrophic lateral sclerosis (ALS) (Maruyama et al., 2010). OPTN is a multifaceted protein and one of its important functions is to protect cells against high levels of oxidative stress by regulating the activation of NF-κB, an important component of the extrinsic apoptotic pathway (De Marco et al., 2006; Zhu et al., 2007; Chalasani et al., 2008). NF-κB is also found in both the cytosol and mitochondrial compartments and its upregulation inhibits cytochrome *c* oxidase and cytochrome *b* mRNA, disrupting the structural integrity of the mitochondrial respiratory chain and adversely affecting ATP synthesis (Chalasani et al., 2007). Furthermore, OPTN co-localises to the Golgi apparatus and it probably plays an important role in membrane trafficking and vesicular transport (del Toro et al., 2009). The neuropathology in ALS is characterised by the degeneration of upper and lower motor neurones, and it is intriguing that *OPTN* mutations seem to have a predilection for long neuronal populations, including RGCs, with mitochondrial dysfunction being one of the likely pathogenetic mechanisms (Shi et al., 2010).

## 8 Demyelination, optic neuritis, and multiple sclerosis

MS is an inflammatory demyelinating disease of the central nervous system and as the disease progresses, extensive axonal loss is observed in the brain and spinal cord (Frohman et al., 2005; Balcer, 2006). The prevalence of MS varies worldwide and in populations of Northern European extraction, the average quoted figure is 1 in 1000 (Fox et al., 2004; Koch-Henriksen and Sorensen, 2010). Up to half of all patients with MS will experience an episode of optic neuritis, and in 15–20% of cases, it is the first manifestation of an underlying demyelinative disorder (Arnold, 2005; Balcer, 2006). Optic neuritis classically presents acutely, with loss of central vision, marked dyschromatopsia, and pain on eye movement. Spontaneous visual recovery usually occurs two to three weeks after the first onset of symptoms and it is nearly complete within the next four weeks. The overall visual prognosis is very good and 1 year after an attack of optic neuritis, more than 90% of patients have recovered their premorbid level of visual acuity (Beck et al., 1993). However, in longitudinal studies of MS patients, including those without a previous history of optic neuritis, progressive RNFL thinning was noted on OCT, clearly indicating a chronic process of subclinical RGC loss (Frohman et al., 2008; Zaveri et al., 2008; Talman et al., 2010).

Female LHON carriers are twice more likely to develop an MS-like illness than male carriers, despite the fact that the risk of visual loss is five times higher among males (Section 3.3.5). We propose two main hypotheses to account for this association. It is possible that subclinical disease in at-risk LHON carriers results in immunogenic mitochondrial peptides being exposed, which trigger or potentiate an autoimmune cascade against oligodendrocytes (Loveland et al., 1990; Chalmers et al., 1995). Conversely, the autoimmune process could be the primary pathological factor and the deleterious consequences for oligodendrocytes are magnified by the presence of an underlying mtDNA defect, the latter putting additional stress on mitochondrial oxidative metabolism (Mahad et al., 2007; Mahad et al., 2009). The optic neuropathy in LHON patients who develop an MS-like illness is invariably severe with poor visual recovery and as such behaves differently from the natural history of acute demyelinative optic neuritis. Presumably, RGC axonal loss in these LHON-MS patients is amplified by dysfunctional oligodendrocytes leading to demyelination or failure of remyelination (Sapey et al., 2001; Mao and Reddy, 2010).

The link between mitochondrial dysfunction and MS is not limited to the primary mtDNA LHON mutations. We recently reported the occurrence of a more disseminated demyelinative process in two independent DOA families with genetically-confirmed *OPA1* mutations. Compound heterozygous *POLG1* mutations have also been recently identified in a 4-year-old child with presumed acute disseminated encephalomyelitis (ADEM) (Harris et al., 2010), and in two patients in their thirties presenting initially with unilateral optic neuritis, which subsequently evolved into clinically-definite MS with classical white matter MRI abnormalities and unmatched CSF oligoclonal bands (Echaniz-Laguna et al., 2010). In both adult MS cases, muscle biopsy revealed numerous COX-negative fibres, with prominent RRFs and multiple mtDNA deletions on long-range PCR analysis.

## 9 Optic nerve head morphology and disease susceptibility

Unaffected LHON carriers have larger optic disc areas compared with affected carriers and normal controls. Among affected LHON carriers, visual recovery was associated with larger vertical but not horizontal disc diameters, and there was a trend towards a better visual outcome with larger optic disc areas (Ramos et al., 2009). About 10% of LHON carriers experience visual loss before the age of 10 years, and in these childhood cases, two patterns of visual loss have been described; an acute presentation similar to the one seen in adults, and a milder variant with a slowly progressive course (Barboni et al., 2006). Compared to the slowly progressive group, children with acute LHON had worse visual acuities, thinner peripapillary RNFL thickness, and significantly smaller optic disc areas. The results from these two studies therefore suggest a possible link between optic disc anatomy and the phenotypic expression of the primary mtDNA LHON mutations. Similar to anterior ischaemic optic neuropathy (AION), a small crowded optic disc could predispose LHON carriers to a higher risk of visual loss (Hayreh 2009a,b). Transient and segmental RNFL swelling have been observed in asymptomatic LHON carriers (Nikoskelainen et al., 1996, 1977), and with the advent of OCT imaging, thickening of the temporal RNFL has been documented more objectively, confirming these earlier clinical observations (Barboni et al., 2005, 2009; Savini et al., 2005; Seo et al., 2009). The swelling of these RGC axons is secondary to axoplasmic stasis and it reflects the compromised bioenergetic state within these neurones, which are struggling to maintain energy-dependent cytoskeletal transport mechanisms (Section 10.3). If the optic nerve axons are congenitally packed within a relatively confined space, under the right circumstances, a compartment syndrome could result, further impeding axonal transport. As the fine capillary network around the lamina cribosa becomes compressed by the surrounding tissue oedema, the resulting ischaemia could set up a vicious cycle, which eventually precipitates the onset of visual loss. The concept of a “disc-at-risk” is also attractive in that it could explain the role of environmental factors such as heavy smoking in increasing the risk of disease conversion among LHON carriers. Long-term heavy smokers are more likely to have atherosclerotic vascular changes and these could further exacerbate a local hypoxic insult at the optic nerve head.

**Fig. 9:**
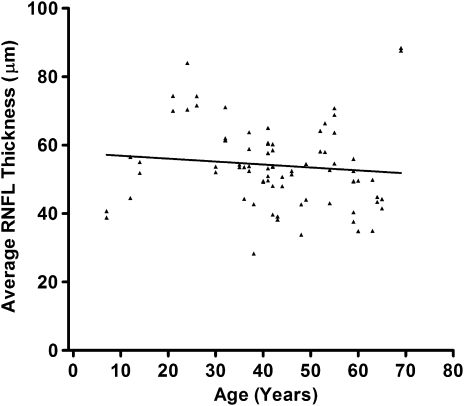
Age-related decrease in average RNFL thickness, consistent with progressive retinal ganglion cell loss among *OPA1* mutational carriers (*n* = 40). Spearman rank correlation coefficient = −0.2419, *P* = 0.0307 (PWYM, unpublished data).

In one study, *OPA1* carriers had significantly smaller optic disc areas compared with normal controls (Barboni et al., 2010), and although speculative, these findings were ascribed to the role played by *OPA1* in early embryonic development (Section 11.3.2). By accentuating developmental apoptosis, *OPA1* mutations could result in a greater loss of RGCs and therefore a correspondingly smaller optic nerve head. In support of this hypothesis, *OPA1* carriers have thinner peripapillary RNFL measurements than controls at all age groups, and extrapolating the data, it is likely that these patients had lower RGC counts at birth (Fig. 9) (Milea et al., 2010). If OPA1 does exert a developmental influence on optic nerve head morphology, this could have important implications for other ocular disorders such as NTG and optic nerve hypoplasia. One could speculate that specific combinations of *OPA1* variants could have a subtle effect on the biomechanical characteristics of the lamina cribosa, which is the principal site of IOP-related damage to RGC axons (Burgoyne et al., 2005; Sigal and Ethier, 2009; Sigal et al., 2010). The mechanical effect of IOP on load-bearing connective tissues is accentuated at the lamina cribosa, and this will vary depending on the unique susceptibility conferred by an individual’s optic nerve head configuration. In individuals with high-risk *OPA1* genotypes and relatively weak optic nerve head biomechanics, axonal damage could occur at relatively low IOPs, increasing the risk of developing NTG. The aetiology of optic nerve hypoplasia is poorly defined and in 20% of cases, this abnormality occurs in isolation, without more widespread cerebral malformations or hypothalamic dysfunction (Borchert and Garcia-Filion, 2008). It would therefore be of great interest to screen for *OPA1* in a cohort of patients with isolated optic nerve hypoplasia to determine whether some patients harbour pathogenic mutations, or whether there is any association with specific polymorphic sequence variants.

## 10 Greater vulnerability of retinal ganglion cells

### 10.1 Anatomical considerations

**Fig. 10:**
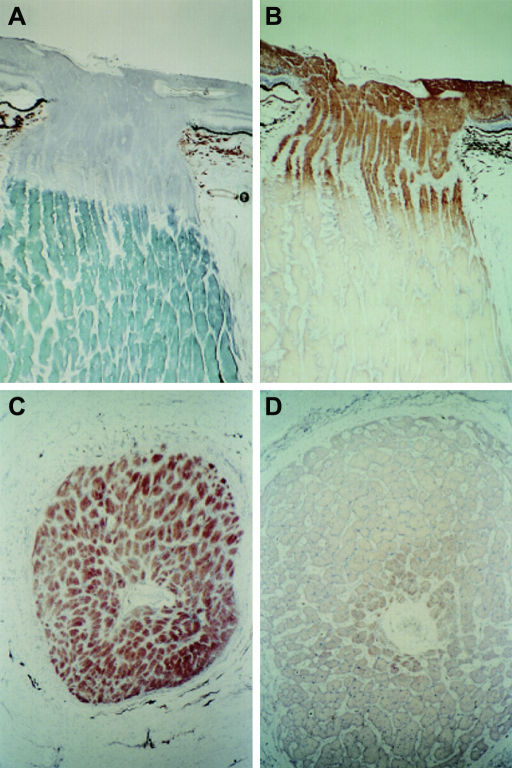
Optic nerve sections stained for myelin and mitochondrial COX activity: (A) Sudan black staining revealing the presence of myelin posterior to the lamina cribosa, (B) marked differential COX activity in the pre- and post-lamina cribosa segments, with intense COX staining in transverse sections taken from the pre-laminar region (C), and significantly lower levels of COX activity in the pos-tlaminar region (D). Reproduced with permission from Andrews et al. (1999a).

**Fig. 11:**
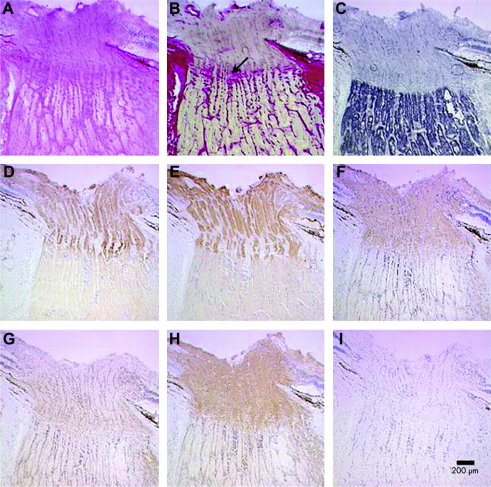
Histology, mitochondrial histochemistry and immunohistochemistry (IHC) performed on serial longitudinal optic nerve sections: (A) haematoxylin and eosin, (B) Van Gieson preparation for connective tissue fibres (Red), with the arrow pointing towards the lamina cribosa, (C) Weigert iron haematoxylin preparation for myelin (Dark blue), (D) COX activity, with a darker stain (Brown) evident in the unmyelinated pre-lamina cribosa segment of the optic nerve, (E) IHC for COX subunit IV revealing a pattern consistent with the level of mitochondrial enzyme activity, (F ) IHC for Na_v_ 1.1 showing a greater concentration of these specific voltage gated Na^+^ channels in the pre-laminar region, (G) IHC for Na_v_ 1.2 demonstrating a uniformly pale labelling pattern in both pre- and post-lamina cribosa areas, (H) IHC for Na_v_ 1.6 with a strong staining reaction observed in the unmyelinated pre-laminar optic nerve, and (I) control optic nerve section with the primary antibody omitted. Reproduced with permission from Barron et al. (2004).

Both *OPA1* and the primary LHON mutations result in a mitochondrial biochemical defect, but the same situation applies to other cell types such as photoreceptors and substantia nigra neurones, which have similar, if not higher, oxidative requirements than RGCs. What other factors therefore contribute to this greater vulnerability of RGCs in mitochondrial optic neuropathies? The optic nerve has some rather peculiar anatomical constraints which could influence the pathological consequences of these genetic defects. One such feature is the fact that RGC axons only acquire a myelin sheath beyond the lamina cribosa. In the pre-laminar region, the generation and propagation of action potentials are therefore less efficient and require greater energetic input (Carelli et al., 2004a; Morgan, 2004). To investigate this further, we performed a series of histological studies of human optic nerves, first staining them for mitochondrial COX and succinate dehydrogenase (SDH) enzyme activities (Andrews et al., 1999a; Bristow et al., 2002). As we expected, the pre-laminar, unmyelinated optic nerve demonstrated much more intense COX staining compared with the post-laminar, myelinated segment, clearly indicating a differential concentration of mitochondria across the lamina cribosa (Fig. 10). The increased mitochondrial content within the unmyelinated RGC axons was associated with a high density of voltage gated sodium channels, reflecting the greater energy requirements for nerve conduction in the absence of myelination (Fig. 11) (Barron et al., 2004). The pre-laminar segment of the optic nerve could therefore be viewed as a vulnerable metabolic “chokepoint”, magnifying the consequences of any impairment in mitochondrial OXPHOS, however subtle. Another important point to consider is that parvocellular RGCs within the papillomacular bundle have relatively small cross-sectional areas, which could put them at an even greater physiological disadvantage in terms of their mitochondrial reserve, compared with the larger magnocellular RGCs (Sadun et al., 2000).

Another cranial nerve marked by such a dramatic transition between unmeylinated and myelinated segments is the cochlear nerve. Nearly two-thirds of all patients with DOA+ phenotypes develop sensorineural deafness (Yu-Wai-Man et al., 2010b). It is therefore very revealing that in two family members with optic atrophy and hearing loss due to a pathogenic *OPA1* mutation, auditory investigations localised the defect to the terminal unmyelinated portions of cochlear nerve (Huang et al., 2009). Transtympanic electrocochleography revealed sub-threshold, abnormally-prolonged negative potentials within the unmyelinated auditory nerve terminals suggesting a failure to generate action potentials at the first node of Ranvier.

### 10.2 Mitochondrial network dynamics

The mitochondrial network is in a constant state of flux and it relies on a delicate balance between fusional and fissional forces. Disruption of mitochondrial fusion can have dramatic consequences on cell survival as clearly illustrated by *OPA1* and *MFN2* mutations. The role of mitochondrial network dynamics in maintaining cellular homeostatis was confirmed further when a heterozygous, dominant-negative, *DLP1* mutation was identified in a female infant with a complex neurodegenerative phenotype characterised by microcephaly, abnormal brain development, optic atrophy, and disturbed metabolic function (Waterham et al., 2007). The mitochondrial network in the patient’s cultured fibroblasts was elongated, forming tangled tubular structures, which were clumped around the nucleus. *DLP1* codes for DRP1, a pro-fission, dynamin-related GTPase protein, and the mutation leads to a dominant-negative loss of function, resulting in unopposed mitochondrial fusion. A dysfunctional mitochondrial network can obviously impact on other cellular processes such as axonal transport, but as such, it still does not explain the selective vulnerability of RGCs. Some important clues were uncovered by Kamei et al. (2005), who transfected cultured rat RGCs and cerebellar granule cells (CGCs) with short interfering RNAs (siRNAs) against *Opa1* transcripts. RGCs were much more susceptible to these *Opa1*-blocking siRNAs and they exhibited a greater degree of mitochondrial fragmentation compared with CGCs, reflecting the predominant optic nerve phenotype seen in patients with DOA.

### 10.3 Mitochondrial–cytoskeletal interactions

**Fig. 12:**
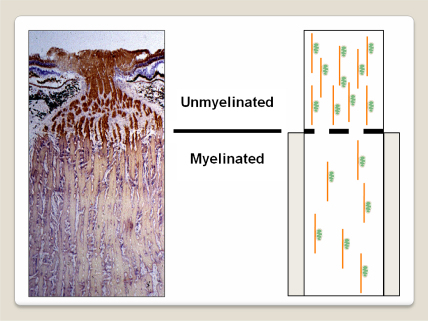
The importance of the cytoskeleton in maintaining the differential concentration of mitochondria in the pre- and post-lamina cribosa segments of the optic nerve. The left panel shows a longitudinal section of a human optic nerve stained sequentially for COX and SDH. The pre-lamina, unmyelinated segment has a much darker COX staining consistent with the higher concentration of mitochondria. The right panel is a schematic representation of the cytoskeletal–mitochondrial interactions which facilitate the transport, distribution, and localisation of mitochondria to different areas of the optic nerve.

Mitochondria do not exist in isolation and an intricate cytoskeletal system is essential for their proper localisation to areas of increased energetic demands (Fig. 12) (Das et al., 2010). These mitochondrial–cytoskeletal interactions apply not only to the lamina cribosa, but also to other sites requiring the maintenance of a differential mitochondrial gradient such as the nodes of Ranvier. Axonal transport along the relatively long RGC axons is mainly dependent on the microtubule tracks, which are powered by the energy-dependent dynein and kinesin family of motor proteins (Yaffe et al., 2003; Boldogh and Pon, 2007). Conceptually, abnormal axonal transport could therefore be disrupted by two processes, either a mitochondrial biochemical defect or a primary problem with the assembly and maintenance of the microtubule network, as seen in the HSP group of disorders (Salinas et al., 2008). The distinction is somewhat artificial in that one will precipitate the other, setting up a vicious circle leading to axonal swelling due to axoplasmic stasis, and ultimately axonal degeneration marked by the onset and progression of clinical symptoms (Morfini et al., 2009).

### 10.4 Light and mitochondrial toxicity

RGCs are constantly being exposed to light in the 400–760 nm wavelength spectra, the cornea and the lens effectively blocking ultraviolet light below 400 nm. The blue component of visible light has the shortest wavelength and in studies of the retinal pigment epithelium and photoreceptors, it had the greatest potential for inducing cellular ROS production. Photoreceptors are partially shielded by macular lutein and zeoxanthin pigments (Chen et al., 2001; Landrum and Bone, 2001), whereas mitochondria within the pre-laminar, unmyelinated RGC axons are directly exposed to light and do not have the benefit of these protective mechanisms. It is therefore possible that chronic light exposure could tip the balance in RGCs already compromised by a genetically-determined mitochondrial respiratory chain defect. This hypothesis has been partially tested in RGC-5 cell lines exposed to incremental light intensities, and cultured in both normal media and conditions of serum deprivation (Lascaratos et al., 2007; Osborne et al., 2008). Light exposure resulted in the generation of a significant amount of ROS in these RGC-5 cells, an effect which was intensity-dependent and exacerbated in conditions of nutritional deprivation. This light-induced elevation in ROS levels affected cellular viability by suppressing the expression of RGC-specific mRNAs and triggering the apoptotic cascade. Additional studies are therefore warranted to investigate the consequences of light exposure on RGC survival in mitochondrial optic nerve disorders, and for that purpose, both patient cell lines and existing animal models could be used (Lascaratos et al., 2007; Osborne et al., 2008).

### 10.5 Melanopsin retinal ganglion cells

Much attention has focused on the selective vulnerability of RGCs in mitochondrial optic neuropathies. However, immunohistochemical analysis of post-mortem eyes from patients with LHON and DOA has revealed relative sparing of a specific subpopulation of melanopsin-containing RGCs (La Morgia et al., 2010). These melanopsin RGCs constitute only about 1% of the total RGC population, but they subserve an important evolutionary role, regulating the body’s circadian rhythm as part of the retino-hypothalamic tract. The relative sparing of these melanopsin RGCs in LHON and DOA also explain to a certain extent the normally preserved pupillary light reflexes in these two disorders. The inherent resistance of melanopsin RGCs to mitochondrial dysfunction is tantalising and it could reveal key protective mechanisms that could be applied to neuroprotection of the larger pool of susceptible RGCs.

## 11 Experimental disease models

### 11.1 *Drosophila dOpa1* mutants

Yarosh et al. (2008) have recently established a *Drosophila* model harbouring a specific *dOpa1* mutation (CG8479), and the ocular phenotypes associated with heterozygous and homozygous carriers were determined. Homozygous mutant flies developed a rough and glossy eye phenotype due to the loss of hexagonal lattice cells, and decreased lens and pigment deposition. The *dOpa1* mutation caused an increase in ROS levels and mitochondrial fragmentation, which damaged both cone and pigment cells. In a series of elegant experiments, these investigators were then able to demonstrate that the rough and glossy eye phenotype could be partially reversed by dietary supplementation with SOD-1 and vitamin E, and by genetic overexpression of human *SOD1*. Heterozygous adult flies did not exhibit any ocular abnormalities, but similar to the homozygous mutants, they also demonstrated elevated ROS levels and a greater susceptibility to oxidative stress. The heteterozygous drosophila carriers showed irregular and dysmorphic mitochondria in their muscle, and they had a significantly shortened lifespan, which was only partially restored by anti-oxidant treatment (Shahrestani et al., 2009; Tang et al., 2009).

### 11.2 Zebrafish *opa3* mutants

High levels of *opa3* mRNA transcripts are present in the central nervous system of zebrafish (*Danio rerio*), including the optic nerve and retinal layers. Zebrafish embryos were therefore engineered harbouring a 5.2 kb retroviral DNA insertion immediately downstream of the mitochondrial leader signal, which disrupts the *opa3* reading frame and results in a premature termination codon (Pei et al., 2010). Five-days-old homozygous mutant embryos (*opa3*^ZM/ZM^) had increased MGA levels, recapitulating the biochemical signature of patients with Costeff syndrome (Section 4.3). A significant loss of RGCs together with a reduction in optic nerve diameter was also observed on histological analysis of 1-year-old *opa3*^ZM/ZM^ zebrafish mutants, confirming the role of *opa3* in preserving RGC function. In addition, these homozygous mutants demonstrated extra-ocular deficits with dramatic alterations in swimming behaviour secondary to ataxia, loss of buoyancy control, and hypokinesia.

### 11.3 Current murine models

#### 11.3.1 LHON

There is still no animal model where the primary LHON mutations have been successfully introduced into the mitochondrial genome. In spite of these technical difficulties, four experimental techniques have been developed which can replicate the optic nerve degeneration seen in LHON: (i) intravitreal injection of a respiratory chain poison such as rotenone (Zhang et al., 2002), (ii) stereotactic injection of biodegradable rotenone-loaded microspheres into the optical layer of the superior colliculus (Marella et al., 2010), (iii) downregulation of nuclear-encoded complex I subunits (e.g. NFUFA1) with specific mRNA-degrading ribozymes (Qi et al., 2003), and (iv) allotopic expression of mutant subunits (e.g. MTND4) which are then imported into the mitochondria (Qi et al., 2007b; Ellouze et al., 2008). These disease models will be indispensable when testing the feasibility of gene therapy in LHON, and different strategies are currently being pursued (Section 12.4).

#### 11.3.2 *Opa1*

Two DOA mouse models have been developed which harbour pathogenic mutations in exon 8 (c.1051C>T) and intron 10 (c.1065+5g>a) of the *Opa1* gene (Alavi et al., 2007; Davies et al., 2007). These mutations are truncative in nature and they result in a 50% reduction in the overall expression of the Opa1 protein. In both models, homozygous mutant mice (*Opa1*−/−) died *in utero* during embryogenesis, highlighting the central role played by the Opa1 protein in early development. Heterozygous *Opa1*+/− mice faithfully replicated the human phenotype exhibiting a slowly progressive optic neuropathy and demonstrating objective reduction in visual function on psychophysical testing. Visual evoked potential (VEP) measurements showed significantly reduced amplitudes, but no change in latencies, supporting an ascending progress of degeneration from the soma towards the axon (Heiduschka et al., 2010). Histological and retrograde labelling experiments confirmed a gradual loss of RGCs and an associated thinning of the retinal nerve fibre layer. The surviving optic nerve axons had an abnormal morphology with swelling, distorted shapes, irregular areas of demyelination and myelin aggregates. These features of optic nerve degeneration were seen as early as 9 months, but they were much more visible by 24 months. Loss of dendritic arborisation was observed for on- but not off-centre RGCs, these early features of neuronal dysfunction preceding the onset of axonal loss (Williams et al., 2010). An increased number of autophagosomes was also noted in the RGC layer of heterozygous *Opa1*+/− mice at later time points, probably due to the accumulation of dysfunctional mitochondria (White et al., 2009). Mitochondria within these axons showed disorganised cristae structures on transmission electron microscopy and cultured fibroblasts showed increased mitochondrial network fragmentation. To further investigate the extent of mitochondrial dysfunction, we carried out additional histochemical and molecular genetic studies on both these DOA mouse models (Yu-Wai-Man et al., 2009a). COX deficiency and multiple mtDNA deletions were not detected in sketelal muscle and the RGC layer of heterozygous mutant mice, implicating other *Opa1*-related disease mechanisms in precipitating optic nerve degeneration.

A third *Opa1* mouse model is currently being characterised harbouring a heterozygous c.2708-2711delTTAG mutation in exon27. Heterozygous mutant mice are showing signs of optic nerve degeneration from the age of 6 months, with subnormal VEPs, and abnormal mitochondrial distribution, especially in the lamina cribosa region (Dr Guy Lenaers, Montpellier, personal communication). All these *Opa1* mouse models only manifest pure optic atrophy, and it will be important to create a DOA+ mouse model in order to dissect the mechanisms which contribute to multi-system organ involvement.

The role played by Opa1 in early mouse embryogenesis was recently confirmed in the *lilR3* mutant mouse where a proportion of embryos was noted to die at midgestation (E11.5) displaying growth retardation, exencephaly, and abnormal patterning along the anterior–posterior axis (Moore et al., 2010). Using a meiotic mapping strategy, Moore et al. (2010) uncovered the genetic basis for this embryonic lethality by identifying a homozygous splice site mutation within intron 19 of the *Opa1* gene, which results in a 6-bp intronic sequence insertion. As a result of this *Opa1* mutation, the mutant Opa1 protein is mislocalised, remaining within the cytosol instead of being properly translocated to the mitochondrial inner membrane.

#### 11.3.3 *Opa3*

An *Opa3* mouse model carrying a c.365T>C (p.L122P) mutation has been reported (Davies et al., 2008). Heterozygous *Opa3*+/− mice were not compromised, whereas homozygous *Opa3*−/− mice developed multi-system organ failure with cachexia, dilated cardiomyopathy, extrapyramidal features, and a reduced lifespan of less than 4 months. These mice had severely impaired visual function, and although all the retinal layers were affected, cell loss was much more prominent within the RGC layer, further reinforcing the selective vulnerability of this specific cell type.

#### 11.3.4 Glaucoma

A series of papers has been reported by the Weinreb group exploring the relationships between Opa1 and IOP-mediated RGC loss in murine glaucoma models (Ju et al., 2008a,b, 2009a,b, 2010). Several interesting observations were made: (i) increased IOPs led to apoptotic RGC loss, which was preceded by mitochondrial network fragmentation, and marked Opa1 and cytochrome *c* release into the cytosol, (ii) the IOP effect was mediated by glutamate receptor activation, which could be blocked by the *N*-methyl-d-aspartate (NMDA) glutamate receptor antagonist memantine, and (iii) overexpression of Opa1 with an adenovirus-associated virus (AAV) vector was protective against RGC death in glaucomatous optic neuropathy.

### 11.4 *In vivo* imaging of retinal ganglion cells

Analysis of fixed retinal whole mount preparations is time-consuming and rather limited given that the animal in question needs to be sacrificed. A better understanding of the complex pathological mechanisms underlying inherited optic neuropathies will therefore require more sophisticated *in vivo* techniques for visualising RGCs, with the need for both time-lapse imaging and long-term serial monitoring of various cellular processes. Apoptosis has been implicated in all mitochondrial optic neuropathies and a non-invasive system which allows RGC death to be visualised directly would be a major research tool. Glaucoma investigators are working on different experimental paradigms, which include (i) retrograde labelling of RGCs, with tracer injection at the superior colliculus (Kobbert et al., 2000; Gray et al., 2008), (ii) transduction of RGCs following intravitreal injection of a recombinant AAV vector carrying GFP (Folliot et al., 2003), and (iii) DARC (Detection of apoptosing retinal cells) with intravitreal injection of fluorescently-labelled annexin 5, which binds to exposed phosphatidylserine in early stages of apoptosis (Cordeiro et al., 2004; Guo and Cordeiro, 2008).

All these apoptosis-imaging techniques are invasive and they require repeated injections, with the possibility of introducing non-disease-related artefacts. To overcome these technical issues, various groups have generated transgenic mice which express a fluorescent marker such as GFP, under a specific RCG promoter such as Thy-1 (Walsh and Quigley, 2008; Leung and Weinreb, 2009). Although further improvement in transfection efficiency is needed, RGCs which are successfully transfected can be visualised using a standard confocal scanning laser ophthalmoscope (CSLO), with the mouse anaesthesised on an appropriately adapted microscope stage. Using this protocol, extremely detailed RGC images can be obtained including cell body morphology and the extent of dendritic arborisation. Furthermore, coupled with the intravitreal injections of tracer agents, time-lapsed sequences would allow axonal transport to be quantified both in the pre- and post-laminar segments of the optic nerve. The application of these new imaging modalities to both existing and future animal models of mitochondrial optic neuropathies will be a major breakthrough, allowing for example early subclinical RGC abnormalities to be detected, and the therapeutic potential of putative neuroprotective agents to be assessed at a more functional level.

## 12 Treatment strategies for inherited optic neuropathies

### 12.1 Genetic counselling

Male carriers harbouring mtDNA point mutations can be reassured that their children are not at risk of inheriting their genetic defect. Female carriers will transmit the mutation to all their offspring, but if the mutation is heteroplasmic, it is not possible to reliably predict the mutational level that will be transmitted. As discussed earlier, significant variations in heteroplasmy levels can occur as a result of the mitochondrial genetic bottleneck. The use of amniocentesis or chorionic villus sampling for prenatal testing is therefore limited in this situation, as the mutational load detected in amniocytes and chorionic villus cells could differ from other foetal tissues, especially those at greater risk from a specific mtDNA point mutation such as RGCs (Brown et al., 2006). Although it is not possible to accurately predict whether a LHON carrier will eventually lose vision, individuals can be counselled based on the two major identifiable risk factors in this disorder, age and gender. Male carriers have about a 50% lifetime risk of visual failure compared with only 10% for female carriers, and most patients will experience visual loss in their late teens and twenties. The probability of becoming affected decreases subsequently and disease conversion in LHON is rare over the age of 50 years. The risks of transmission for nuclear mitochondrial disorders follow the laws of Mendelian inheritance, but if a specific nuclear defect has not been identified, only an approximate risk can be provided based on the family history. As a result of the marked inter- and intra-familial phenotypic variability seen with both pure and syndromal inherited optic neuropathies, genetic counselling for patients and their families remains a challenging area of practice.

### 12.2 Supportive measures

The treatment options for patients with inherited optic neuropathies are currently limited (Chinnery et al., 2006). However, there are several practical steps that can be taken as part of a multi-disciplinary team to improve a patient’s quality of life, and minimise long-term morbidity (Yu-Wai-Man et al., 2009b; Fraser et al., 2010). Depending on their needs, these patients should be provided with access to facilities such as low visual aids and occupational therapy, and clinicians can help with financial assistance through their local social services. It is also important to aggressively manage related medical problems such as diabetes and epilepsy, and clinicians need to be vigilant to the development of new complications such as cardiomyopathy and sensorineural deafness, which could be amenable to therapeutic intervention. Patients with mitochondrial disorders should be strongly advised not to smoke and to minimise their alcohol intake, not only as a general health measure, but smoking, and to a lesser extent excessive alcohol intake, have been linked with an increased risk of visual loss among LHON carriers (Kirkman et al., 2009b). Patients with CPEO often get significant benefit from simple conservative measures such as ptosis props or Fresnel prisms for symptomatic ocular misalignment (Richardson et al., 2005). In some cases, strabismus and ptosis surgery are indicated but these should be performed by experienced surgeons (Shorr et al., 1987; Ahn et al., 2008), because of the increased risk of complications such as corneal exposure secondary to poor orbicularis oculi function and impaired Bell’s phenomenon (Daut et al., 2000).

### 12.3 Neuroprotection

On the basis of limited, mostly anecdotal evidence, various combinations of pharmacological agents have been used to treat patients with mitochondrial disorders including multi-vitamin supplements, co-enzyme Q_10_ (CoQ_10_) and its derivatives, and putative free radical scavengers (Horvath et al., 2008; Tarnopolsky, 2008; Schon et al., 2010). There is relatively more clinical data on the use of CoQ_10_, which has shown a clear benefit for patients with primary CoQ_10_ deficiency. In one randomised, double-blind, crossover study of 16 patients with mitochondrial cytopathies, a combination of CoQ_10_ with creatine monohydrate and α-lipoic acid, reduced the levels of resting plasma lactate and oxidative stress markers (Rodriguez et al., 2007).

Idebenone is a synthetic analogue of CoQ_10_, and it is currently being investigated as a treatment option for LHON and other neurodegenerative disorders such as FRDA. Idebenone is able to operate under low oxygen tension and it is thought to have anti-oxidant properties, in addition to optimising ATP production by the respiratory chain complexes (Tonon and Lodi, 2008; Sacconi et al., 2010). Initial reports of idebenone therapy in FRDA showed promising results with an improvement in both cardiac and neurological status (Rustin et al., 2004; Di Prospero et al., 2007). However, preliminary data released from two recently completed randomised controlled trials have proven rather disappointing, with no significant difference in primary cardiac and neurological endpoints between the idebenone-treated and placebo groups. (http://www.santhera.com/index.php?docid=212&vid=&lang=en&newsdate=201005&newsid=1417424&newslang=en, Accessed 1st of December 2010). In a retrospective study of 28 affected LHON patients, half of whom received a combination of idebenone, vitamin B2, and vitamin C for at least 1 year, the treated group showed a faster rate of visual recovery (Mashima et al., 2000). However, in a more recent report, megadoses of idebenone, vitamin C, and riboflavin did not prevent second-eye involvement in two m.11778G>A LHON carriers treated after the onset of unilateral visual loss, and both patients showed no improvement in visual function (Barnils et al., 2007). The benefits of idebenone in LHON therefore remain unclear, but being a safe drug, it is often recommended by clinicians or self-prescribed by patients. In collaboration with clinical partners in Germany and Canada, we recently completed a double-blind randomised placebo-controlled trial of idebenone in LHON. The preliminary results suggest a beneficial effect, especially among patients with recent disease onset, and relatively good remaining visual acuity in the second eye to become affected. (http://www.santhera.com/index.php?docid=212&vid=&lang=enamp;newsdate=201006&newsid=1424223&newslang=en, Accessed 1st of December 2010). If these initial findings are confirmed, idebenone could also be considered as a neuroprotective agent for other mitochondrial optic neuropathies such as DOA, although long-term follow-up will be required given the relatively slower rate of RGC loss in this disorder.

Brimonidine is a topical α-2 agonist used in the management of glaucoma for its IOP-lowering properties. In addition, studies based on models of optic nerve ischaemia have suggested that brimonidine could have an anti-apoptotic effect, mitigating RGC loss (Wheeler et al., 2003). On this basis, topical brimonidine has been tested as prophylactic agent for second-eye involvement in an open-labelled study of 9 patients with unilateral acute vision from LHON (Newman et al., 2005). Brimonidine failed to prevent fellow eye involvement and there was no evidence of improved visual benefit following the onset of visual loss. In the long term, neuroprotection for LHON remains an attractive treatment option given the easy accessibility of ocular tissues to various forms of manipulation. Various agents have been shown to have RGC-neuroprotective properties in other models of optic nerve degeneration such as memantine, valproic acid (Biermann et al., 2010), prostaglandin J2 (PGJ2) (Touitou et al., 2010), and SIRT-1 activators (Shindler et al., 2007). It is not inconceivable an effective agent will soon become available in clinical practice, which could be safely delivered intravitreally, providing an instantaneous local protective effect.

### 12.4 Gene therapy

Classical gene therapy is proving very challenging for primary mitochondrial disorders, because the tools required for successful gene transfer into the mitochondrial genome is still not available (DiMauro and Mancuso, 2007; Kyriakouli et al., 2008; DiMauro and Rustin, 2009). The mitochondrial inner membrane represents a significant physical barrier that needs to be overcomed, and given the large number of mitochondria per cell, a highly efficient vector will be required to achieve an adequate level of transfection. To bypass these difficulties, allotopic strategies have been developed where the gene of interest is transfected into the nuclear genome, usually with an AAV vector (Manfredi et al., 2002; Qi et al., 2007a). The protein product has a mitochondrial targeting sequence and as a result, it gets imported into the mitochondrial compartment, either replacing the missing protein or complementing the dysfunctional mutant protein.

In both *in vitro* and *in vivo* experimental LHON models, RGC loss was dramatically reduced by transfecting them with an AAV vector containing the human *SOD2* gene (Qi et al., 2004, 2007a). The increased expression of the superoxide dismutase enzyme is thought to improve RGC survival by minimising free radical damage, and decreasing the cell’s susceptiblity to apoptosis. Allotopic rescue, with the replacement of the defective mitochondrial complex subunit, is another attractive option for gene therapy in LHON. Proof-of-principle for this approach has been demonstrated in a rat model expressing a defective *ND4* gene containing the m.11778A>G primary LHON mutation (Ellouze et al., 2008). The loss of visual function in these rats was reversed by transfecting RGCs with the wild-type *ND4* gene, using an *in vivo* electroporation technique instead of an AAV vector. The transgene became stably integrated within the nuclear genome and the level of expression achieved was sufficient for successful RGC neuroprotection. These early studies of allotopic rescue in LHON are promising, but the results need to be replicated in larger animals, and long-term safety data is essential before human clinical trials can be contemplated (Friedmann, 2000; Miller, 2008). The same caveats will apply to proposed gene therapy strategies for nuclear mitochondrial disorders. Issues of safety and efficacy are especially relevant given the significant concerns that have been raised recently about the limitations of allotopic rescue in the treatment of primary mtDNA disorders (Figueroa-Martinez et al., 2010; Perales-Clemente et al., 2010). As yet, there is no conclusive experimental evidence that allotopically-expressed mitochondrial subunits can be properly integrated into fully-assembled OXPHOS complexes. Mitochondrially-encoded complex I subunits are also highly hydrophobic and if a proportion of these polypeptides is not imported, they could have deleterious consequences by physically aggregating into the cytosol or triggering an inappropriate immune response.

### 12.5 Preventing transmission of pathogenic mutations

Following successful fertilisation, two distinct structures can be observed within the fertilised oocyte, known as the male and female pronuclei (Brown et al., 2006). Given the lack of treatment for mitochondrial disorders, pronuclear transfer is currently being investigated as a method to prevent the transmission of mtDNA mutations in human embryos (Tachibana et al., 2009; Craven et al., 2010). This strategy involves the replacement of the entire mitochondrial population, by transferring karyoplast containing the pronuclei from a donor zygote to an enucleated recipient zygote. In a landmark study using abnormally-fertilised human zygotes, Craven et al. (2010) have shown that pronuclear transfer resulted in minimal carry-over of donor zygote mtDNA, and it was compatible with successful progression to the blastocyst stage. Preimplantation genetic diagnosis for nuclear defects is well established and it does not involve the same degree of complexity faced with mtDNA mutations.

## 13 Future directions

The past decade has seen significant advances in the way both clinicians and basic scientists approach inherited optic neuropathies. With greater access to molecular genetic testing, the phenotypic spectrum of classical optic nerve disorders such as LHON and DOA has expanded to encompass a much wider range of clinical features, some overlapping with other neurodegenerative disorders such as CMT, HSP, and even MS. Mitochondrial optic neuropathies could therefore be used as models to understand key pathophysiological aspects of these complex disorders, the optic nerve being relatively accessible and amenable to direct measurements with non-invasive imaging modalities. A fundamental change in our understanding is the realisation that in a significant number of genetically-determined optic nerve disorders, including glaucoma, mitochondrial dysfunction is likely to be part of a final common pathway mediating RGC loss. However, there are still more questions than answers – What combination of secondary factors will eventually prove to be the basis for the marked incomplete penetrance and male bias in LHON? What disease mechanisms explain multi-system tissue involvement in *OPA1* carriers with DOA+ phenotypes, when other family members harbouring the same mutation only have pure optic nerve involvement? Conversely, why is it that only some patients with *MFN2* and *SPG7* mutations develop visual failure and optic atrophy? With rapid advances in genetic technology, bioinformatics, and molecular biology, the next decade will be an exciting time in this area of research. We are hopeful that these future breakthroughs will translate into much-needed, effective treatment strategies for LHON, DOA, and other mitochondrial optic neuropathies.

## Conflicts of interest

None of the authors have any financial interests to disclose.

## Figures and Tables

**Table 2:** Pathogenic mtDNA LHON mutations.

	Mutation	Gene	References
Common	m.3460G>A	*MTND1*	(Howell et al., 1991a; Huoponen et al., 1991)
	m.11778G>A	*MTND4*	(Wallace et al., 1988)
	m.14484T>C	*MTND6*	(Johns et al., 1992; Mackey and Howell, 1992)

Rare	m.3376G>A	*MTND1*	(Blakely et al., 2005)
	m.3635G>A		(Brown et al., 2001)
	m.3697G>A		(Spruijt et al., 2007)
	m.3700G>A		(Fauser et al., 2002b)
	m.3733G>A^a^		(Valentino et al., 2004)
	m.4025C>T		(Huoponen et al., 1993)
	m.4160T>C		(Howell et al., 1991b)
	m.4171C>A^a^		(Kim et al., 2002)
	m.4640C>A	*MTND2*	(Brown et al., 2001)
	m.5244G>A		(Brown et al., 1995)
	m.10237T>C	*MTND3*	(Horvath et al., 2002)
	m.11696G>A	*MTND4*	(De Vries et al., 1996)
	m.11253T>C		(Leo-Kottler et al., 2002)
	m.10663T>C^a^	*MTND4L*	(Brown et al., 2002)
	m.12811T>C	*MTND5*	(Huoponen et al., 1993)
	m.12848C>T		(Mayorov et al., 2005)
	m.13637A>G		(Huoponen et al., 1993)
	m.13730G>A		(Howell et al., 1993)
	m.14325T>C	*MTND6*	(Howell et al., 2003)
	m.14568C>T		(Besch et al., 1999)
	m.14459G>A^a^		(Jun et al., 1994; Gropman et al., 2004; Tarnopolsky et al., 2004)
	m.14729G>A		(Zhadanov et al., 2005)
	m.14482C>G/A^a^		(Howell and Mackey, 1998; Valentino et al., 2002)
	m.14495A>G^a^		(Chinnery et al., 2001b)
	m.14498C>T		(Wissinger et al., 1997)
	m.14568C>T^a^		(Wissinger et al., 1997; Fauser et al., 2002a)
	m.14596A>T		(De Vries et al., 1996)
	m.9101T>C	*MTATP6*	(Lamminen et al., 1995)
	m.9804G>A	*MTCO3*	(Johns and Neufeld, 1993; Howell et al., 2003)
	m.14831G>A	*MTCYB*	(Fauser et al., 2002b)

a These mtDNA mutations are definitely pathogenic. They have been confirmed in ≥2 independent LHON pedigrees and show segregation with affected disease status.

**Table 3:** Biochemical Consequences of the Primary LHON mutations.

MtDNA Mutation	*In Vitro*^a^			*In Vivo*^b^
	Complex I Activity (%)	Respiratory Rate (%)	ATP Synthesis (%)	^31^P-MRS (%)
m.3460G>A	60–80	30–35	90	0–40
m.11778G>A	0–50	30–50	35	75
m.14484T>C	0–65	10–20	90	50

% Decrease relative to controls

a (Parker et al., 1989; Larsson et al., 1991; Majander et al., 1991; Degli Esposti et al., 1994; Smith et al., 1994; Cock et al., 1995, 1998, 1999; Montagna et al., 1995; Oostra et al., 1995; Vergani et al., 1995; Hofhaus et al., 1996; Majander et al., 1996; Carelli et al., 1997; Brown et al., 2000; Baracca et al., 2005)

b (Barbiroli et al., 1995; Cortelli et al., 1997; Lodi et al., 1997, 2000, 2002)

**Table 4:** Primary inherited optic nerve disorders – Pattern of inheritance, reported loci, and causative genes.

Inheritance	Locus	Gene	OMIM	Phenotypes	References
AD	3q28-q29	*OPA1*	165500	Isolated optic atrophy and syndromal forms of dominant optic atrophy (DOA+)	(Delettre et al., 2000; Alexander et al., 2000; Yu-Wai-Man et al., 2010b)
	19q13.2-q13.3	*OPA3*	165300	Optic atrophy and premature cataracts (ADOAC)	(Garcin et al., 1961; Reynier et al., 2004)
	18q12.2-q12.3	*OPA4* (Unknown)	605293	Optic atrophy	(Kerrison et al., 1999)
	22q12.1-q13.1	*OPA5* (Unknown)	610708	Optic atrophy	(Barbet et al., 2005)
	16q21-q22	*OPA8* (Unknown)	–	Optic atrophy and sensorineural deafness	(Carelli et al., 2007c)
AR	19q13.2-q13.3	*OPA3*	606580	3-methylglutaconic aciduria type III (Costeff syndrome)	(Anikster et al., 2001)
	8q21-q22	*OPA6* (Unknown)	258500	Optic atrophy	(Barbet et al., 2003)
	11q14.1-q21	*OPA7* (TMEM126A)	612989	Optic atrophy	(Hanein et al., 2009)
XL	Xp11.4-p11.21	*OPA2* (Unknown)	311050	Optic atrophy	(Assink et al., 1997; Katz et al., 2006)
Mitochondrial	–	–	53500	Leber hereditary optic neuropathy and overlap mitochondrial syndromes	Table 1
